# Examining Public Sentiments and Attitudes Toward COVID-19 Vaccination: Infoveillance Study Using Twitter Posts

**DOI:** 10.2196/33909

**Published:** 2022-04-15

**Authors:** Ranganathan Chandrasekaran, Rashi Desai, Harsh Shah, Vivek Kumar, Evangelos Moustakas

**Affiliations:** 1 Department of Information and Decision Sciences University of Illinois at Chicago Chicago, IL United States; 2 Middlesex University Dubai United Arab Emirates

**Keywords:** coronavirus, infoveillance, COVID-19, vaccination, social media, Twitter study, text mining, sentiment analysis, topic modeling, tweets, content analysis

## Abstract

**Background:**

A global rollout of vaccinations is currently underway to mitigate and protect people from the COVID-19 pandemic. Several individuals have been using social media platforms such as Twitter as an outlet to express their feelings, concerns, and opinions about COVID-19 vaccines and vaccination programs. This study examined COVID-19 vaccine–related tweets from January 1, 2020, to April 30, 2021, to uncover the topics, themes, and variations in sentiments of public Twitter users.

**Objective:**

The aim of this study was to examine key themes and topics from COVID-19 vaccine–related English tweets posted by individuals, and to explore the trends and variations in public opinions and sentiments.

**Methods:**

We gathered and assessed a corpus of 2.94 million COVID-19 vaccine–related tweets made by 1.2 million individuals. We used CoreX topic modeling to explore the themes and topics underlying the tweets, and used VADER sentiment analysis to compute sentiment scores and examine weekly trends. We also performed qualitative content analysis of the top three topics pertaining to COVID-19 vaccination.

**Results:**

Topic modeling yielded 16 topics that were grouped into 6 broader themes underlying the COVID-19 vaccination tweets. The most tweeted topic about COVID-19 vaccination was related to vaccination policy, specifically whether vaccines needed to be mandated or optional (13.94%), followed by vaccine hesitancy (12.63%) and postvaccination symptoms and effects (10.44%) Average compound sentiment scores were negative throughout the 16 weeks for the topics *postvaccination symptoms and side effects* and *hoax/conspiracy*. However, consistent positive sentiment scores were observed for the topics *vaccination disclosure*, *vaccine efficacy*, *clinical trials and approvals*, *affordability*, *regulation*, *distribution and shortage*, *travel*, *appointment and scheduling*, *vaccination sites*, *advocacy*, *opinion leaders and endorsement*, and *gratitude toward health care workers*. Reversal in sentiment scores in a few weeks was observed for the topics *vaccination eligibility* and *hesitancy*.

**Conclusions:**

Identification of dominant themes, topics, sentiments, and changing trends about COVID-19 vaccination can aid governments and health care agencies to frame appropriate vaccination programs, policies, and rollouts.

## Introduction

Since the outbreak of COVID-19, caused by the SARS-CoV-2 virus, in November 2019, the pandemic continues to pose a serious threat to the lives of millions of individuals around the globe. By June 2021, the virus had infected over 176 million individuals, resulting in over 3.8 million deaths worldwide [1]. The impacts of the pandemic on the world economy, well-being, and social norms of daily living have been profound. In light of the threats posed by this virus, scientists have been racing to understand the nature of the virus and discover potential treatment regimens and therapeutic mechanisms to deal with it. Although lockdowns, social distancing, and wearing masks have been the primary measures to control the spread of the virus, effective vaccination is likely to constitute a definitive long-term strategy that can contain the pandemic and help humankind return to normal life [2]. The foreseeable long-term solution to the COVID-19 pandemic is a globally rolled out, safe vaccination program covering substantial portions of the world population. Vaccines can provide both direct protection by minimizing susceptibility to the virus among the uninfected and indirect protection by reducing spread of the virus among those infected [3]. Therefore, development and deployment of vaccines have become a central component in the global strategy to control and mitigate the spread of COVID-19 with several billions of dollars spent in research and development of the vaccines [4]. In December 2020, US regulatory authorities granted emergency and full authorization for vaccines developed by BioNTech and Pfizer, and Moderna and National Institutes of Health. In August 2021, the US Food and Drug Administration (FDA) provided approval for the Pfizer/BioNTech vaccine. Other vaccines that have been granted approvals include those developed by University of Oxford and AstraZeneca, Johnson & Johnson, Sinopharm, Sputnik-V, and Covaxin, among others. Close to 300 vaccines are currently in different phases of development to tackle the virus and its variants [5,6]. Governments across the world are devising strategies to quickly produce, procure, and distribute vaccines to their citizens [7-9].

Social media platforms have become an important conduit and rich source of data for assessing public attitudes and behaviors during health emergencies. In light of the lockdowns and restrictions imposed due to the COVID-19 pandemic, social media platforms have emerged as key forums for the public to express their opinions and experiences pertaining to the pandemic and vaccinations. Examination of social media data could reveal significant trends, patterns, and changes, and can thus serve as a tool for health surveillance and monitoring the trends. This study builds upon the extant infoveillance research on the COVID-19 pandemic by focusing on the discourse pertaining to COVID-19 vaccinations in Twitter. We analyzed over 2.94 million tweets from January 1, 2021, to April 30, 2021, to explore the trends, sentiments, and key themes pertaining to COVID-19 vaccinations.

There is growing interest in understanding public attitudes and opinions about COVID-19 vaccinations. Studies have found vaccine hesitancy to be prevalent globally across multiple countries, although there is some preliminary evidence about lower levels of hesitancy in lower- and middle-income countries as compared to developed nations such as the United States [10-12]. A number of studies have employed surveys to examine public willingness, acceptance, and hesitancy toward COVID-19 vaccines [13-19]. These studies have used responses from 100 to a few thousand respondents, often from a specific country or region. An alternate *infoveillance* approach using social media data has become a complementary, powerful mechanism to understand and explore public attitudes toward COVID-19 vaccination. A summary of studies using social media data to explore COVID-19 vaccines is provided in Table 1.

The extant studies have collectively helped us to uncover some key public concerns and trends regarding vaccinations, vaccine advocacy, and hesitancy. However, most of the existing studies have used data from early periods of the COVID-19 pandemic or initial phases of vaccination. Some of these studies have also not differentiated if the source of a tweet is an individual or an organization. Several thousands of tweets are typically made by news outlets, health agencies, or other organizations. From an infoveillance perspective, it is critical to examine the social media discourses pertaining to COVID-19 vaccines by the common public rather than by news agencies or other organizations. Building upon the emerging body of research, our study differs from this prior research in the following ways. First, we focused on tweets made between January and April 2021, capturing public attitudes during active periods of vaccinations in many countries. Second, we examined English-language tweets from all over the world, without restriction to a region or a country. Third, we focused on tweets made by individuals only, thus capturing public sentiments and concerns. Our study is uniquely positioned and differs from many other similar studies listed in Table 1, as we capture and use the tweets made by the general public, excluding those made by news outlets and other organizations. Fourth, we used advanced text-mining and topic-modeling techniques to unearth themes and topics underlying the Twitter discourse on COVID-19 vaccinations.

**Table 1:** Summary of key studies on COVID-19 vaccines using social media data.

Source	Data set	Time period	Key findings	Limitations/remarks
Yin et al [20]	1.75 million Weibo messages from China	January to October 2020	Identified public opinions pertaining to pricing, side effects, and inactivated vaccines	Restricted to Chinese-speaking Weibo users, including residents of China and those living abroad. The study used posts from verified users.
Hussain et al [21]	23,571 Facebook posts from the United Kingdom and 144,864 from the United States; 40,268 tweets from the United Kingdom and 98,385 from the United States	March 1 to November 22, 2020	Overall averaged positive, negative, and neutral sentiments were at 58%, 22%, and 17% in the United Kingdom, in contrast to 56%, 24%, and 18% in the United States, respectively. Public optimism regarding vaccine development, effectiveness, clinical trials, concerns over their safety, economic viability, and corporation control were identified.	Geographical scope included the United Kingdom and the United States. The study does not mention excluding tweets made by organizations and news outlets.
Guntuku et al [22]	4 million tweets originating from 2957 US counties	December 1, 2020, to February 28, 2021	Topics identified include side effects, conspiracy theories, trust issues in the US health care system in December 2020; mask wearing, herd immunity, natural infection, and concerns about nursing home residents and workers in January 2021; and access to black communities, vaccine appointments, family safety, and online misinformation campaigns in February 2021. Geographic variations on the topics across different counties were also identified.	Geographical scope was restricted to the United States. The study does not mention excluding tweets made by organizations and news outlets.
Bonnevie et al [23]	1,438,251 tweets; 6498 per day	Antivaccine tweets from February 15, 2020, to June 14, 2020, as compared to those in the pre-COVID-19 period of October 15, 2019, to February 14, 2020	Mentions of vaccine opposition increased by 79.9%. The themes identified were negative health impacts, pharmaceutical industry, policies and politics, vaccine ingredients, federal health authorities, research and clinical trials, religion, vaccine safety, disease prevalence, school, and family	No mention of exclusion of tweets made by organizations and news outlets
Griffith et al [24]	3915 tweets about vaccine hesitancy from Canada	December 10, 2020, to December 23, 2020	Vaccine hesitancy was attributed to the following themes: concerns over safety, suspicion about political or economic forces driving the COVID-19 pandemic or vaccine development, a lack of knowledge about the vaccine, antivaccine or confusing messages from authority figures, and a lack of legal liability from vaccine companies	Geographical scope restricted to Canada, with limited sample size; manual coding of tweets
Hou et al [25]	7032 tweets and Weibo posts from five locations: New York, London, Mumbai, Sao Paulo, and Beijing	June and July 2020	Beijing users (76.8%) had a higher vaccine acceptance rate as compared to those in New York (36.4%). Concerns expressed included: vaccine safety, distrust in governments and experts, widespread misinformation, vaccine production and supply, vaccine distribution, and inequity	Manual coding of tweets and Weibo posts from five locations, with limited sample size. However, this study excluded posts from news outlets and organizational accounts
Yousefinaghani et al [26]	4,552,652 tweets about COVID-19 vaccines	January 2020 to January 2021	Sentiment analysis revealed positive being the dominant polarity and having higher engagement. Themes among the positive-sentiment tweets were happiness and hope, support, and religion. Themes among the negative-sentiment tweets were fear and frustration, disappointment, anger, and politics. More discussion on vaccine rejection and hesitancy as compared to provaccine themes	Examined tweets from six countries: the United States, the United Kingdom India, Australia, Canada, and Ireland. No mention of excluding organizational tweets.
Hu et al [27]	308,755 geo-coded tweets from the United States	March 1, 2020, to February 28, 2021	Identified three phases along the pandemic timeline and documented changes in public sentiments and emotions. An increase in positive sentiment coupled with a decrease in negative sentiment concerning vaccines were noted in most states. Major international or social events and announcements by influential leaders or authorities associated with changes in public opinions toward vaccines.	Geographical scope restricted to the United States. No mention of excluding organizational tweets
Lyu et al [28]	1,499,421 tweets	March 11, 2020, to January 31, 2021	16 topics under five broad themes were identified: opinions and emotions around vaccines and vaccination, knowledge around vaccines and vaccination, vaccines as a global issue, vaccine administration, and progress on vaccine development and authorization	Did not exclude organizational tweets, but eliminated tweets by bots and fake accounts
Eibensteiner et al [29]	Poll of 3439 Twitter users	February 12, 2021, and February 19, 2021	45.9% of Twitter users felt the safety of the COVID-19 vaccines to be adequate; over 82.8% responded affirmatively about taking the vaccination	Used an anonymized polling/survey method with a limited sample of Twitter users

In this research, we sought to uncover important themes underlying the social media discourse pertaining to COVID-19 vaccinations. This will help us to better understand how individuals feel about COVID-19 vaccinations, their inclinations for uptake, as well as reasons behind their hesitancy. Given the prevalence of vaccine hesitancy worldwide [30], it is important to understand public attitudes toward vaccines, underlying reasons for hesitancy, and individual experiences with vaccinations. Moreover, it is also important to uncover how the public feels about various governmental- and policy-related measures that various governments across the world have taken regarding COVID-19 vaccines [31,32]. Using topic modeling and text mining, we seek to uncover the trends and themes underlying social media discourse about COVID-19 vaccinations. A deeper understanding of specific themes and topics can help to frame better responses toward COVID-19 vaccination campaigns and can help policymakers and health professionals in their efforts to improve vaccine uptake.

Our specific research goals were to (1) explore the themes and topics underlying social media discourse pertaining to COVID-19 vaccines and (2) uncover trends and temporal variations in sentiments underlying COVID-19 vaccine discourse in Twitter.

## Methods

### Data Set and Ethical Considerations

This study used publicly available and accessible tweets made by individuals on the Twitter platform, which formed the data set used for our analysis. We present our analysis in aggregate form without identifying specific individuals who made the Twitter posts. Therefore, the activities described do not meet the requirements of human subjects research and did not require review by an institutional review board.

### Data Gathering

We used the Python scraper *snscrape* to collect historical tweets regarding COVID-19 vaccines and vaccination [33]. Our search terms included a combination of “vaccine” and COVID-19–related terms (“covid,” “coronavirus,” “covid19,” “covid-19,” “ncov2019,” and “SARS-CoV-2”) to retrieve tweets published between January 1, 2021, and April 30, 2021. Snscrape and Getoldtweets are popular Python libraries that have been used in several infoveillance studies to capture Twitter data [26,34,35]. We ensured removal of retweets and duplicates so that the data set contained only the original tweets made by the users.

### Data Preprocessing

We used a machine learning approach to separate tweets made by individuals and organizations. Following the approach outlined by Chandrasekaran et al [35], we developed a naive-Bayes classifier to distinguish the Twitter user as being an individual or an organization. The accuracy was 91.81%, providing confidence about the classifier that we used to segregate tweets made by individuals.

Our next step involved preprocessing and cleaning of tweets using a set of libraries in Python. Using the *re*, *nltk*, and *sklearn* libraries, we removed punctuations, stop words, and emojis, and also lemmatized the text of tweets to prepare them for further processing.

### Topic Modeling and Sentiment Analysis

Topic modeling is an unsupervised machine learning method for identifying latent patterns of words in a large collection of documents. The most representative method for topic modeling is latent Dirichlet allocation (LDA), which is a generative probabilistic method [36]. LDA does not assume any prior knowledge of topics, and through appropriate tuning of parameters, one can explore different topic formations and clusters [37]. Often, LDA can simply generate topics that can neither be meaningful nor effective. To overcome the restrictions and limitations of LDA, newer algorithms such as Correlation Explanation (CorEx) have been developed [38]. The CorEx model, similar to LDA, does not make any assumptions about topics in the underlying data. Further, CorEx identifies latent topics that are maximally informative about a collection of documents by examining how words are used in tweets and picks up on patterns to assess what the tweets convey. CorEx allows a researcher to iterate with different numbers of topics, review them, and identify the optimal number of topics for further assessment. CorEx has been effectively used in a number of health infoveillance studies to uncover topics in Twitter data [39,40].

We used CorEx and iterated with a varying number of topics (eg, 5, 10,15, 20, 30). The keywords for different topics were assessed by the authors to ascertain their coherence and meaningfulness pertaining to a topic. The total correlation scores were compared across iterations to decide on the optimal number of topics produced. Next, we reviewed the results to infer appropriate topics on the basis of keywords. We also examined a set of randomly chosen tweets for each topic to assess if those tweets were consistent with the topic. Through discussions, the authors then grouped the topics into broader themes. Our procedures are consistent with similar studies that have examined social media data using text mining and topic modeling [35,39]. Further, we also computed the sentiment score for each tweet using the VADER (valence aware dictionary and sentiment reasoner) tool in Python. VADER is a lexicon and rule-based sentiment analysis tool that is appropriate for social media texts such as tweets [41]. VADER’s polarity score quantifies the sentiment of a tweet in the range from –1 (extreme negative) to 1 (extreme positive). VADER’s scoring method takes into account both the polarity and the intensity of emotion expressed in a tweet. The VADER output labels each tweet into one of the following five sentiments: overly positive (polarity score≥0.70), positive (polarity score between 0.01 and 0.70), neutral (polarity score between –0.01 and 0.01), negative (polarity score between –0.01 and –0.70), and overly negative (polarity score≤–0.70). We used the polarity score to classify the sentiment in the tweets.

In addition to topic modeling and sentiment analysis, we also performed qualitative analysis of tweets in each theme/topic to obtain further insights and temporal trends in the vaccine-related tweets.

## Results

### Tweets Retrieved

Our data gathering resulted in an initial set of 3,707,187 tweets. We removed 762,657 tweets made by organizations. Consistent with our research goal of assessing public sentiments and attitudes, 2,944,530 tweets made by 1,210,225 Twitter users were included in our analysis.

**Figure 1:**
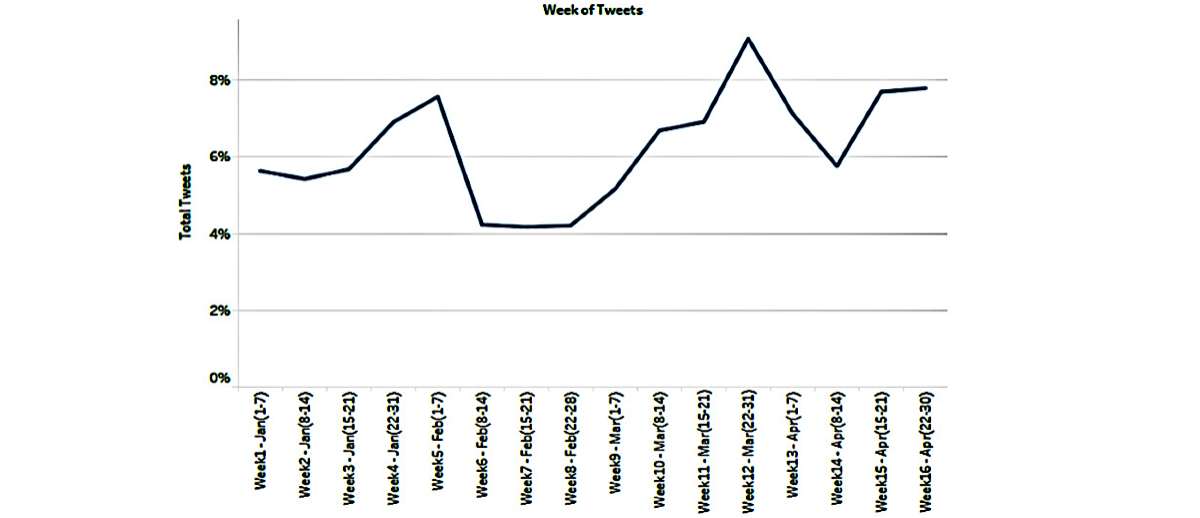
Proportion of COVID-19 vaccine–related tweets from January to April 2021.

The trends in the number of tweets about COVID-19 vaccines from January to April 2021 are presented in Figure 1. All of the weeks had over 100,000 tweets; however, a spike in the number of tweets was observed in the week of March 22-31, 2021. This was the week when the eligibility for receiving COVID-19 vaccines was changed to cover several individuals and groups with several US states opening up vaccination to larger sets of individuals.

### Themes and Topics

Our CoreX topic modeling resulted in 16 topics (Table 2), which were further categorized into six broad themes: *vaccination experiences* (17.27%), *pharma industry (vaccine development, production, and distribution)* (15.71%), *vaccination policies* (21.42%), *vaccination rollout* (5.99%), *attitudes toward vaccination* (37.12%), and *gratitude toward health care workers* (2.49%). The topics and representative keywords are shown in Multimedia Appendix 1. The top three topics that were tweeted in the January to April 2021 timeframe were: *regulatory issues (mandatory vs optional)* (13.94%), *vaccine hesitancy* (12.63%), and *postvaccination symptoms and side effects* (10.44%).

**Table 2:** Topics and broad themes underlying COVID-19 vaccine–related tweets (N=2,944,530).

Themes and topics		Tweets, n (%)
**Vaccination experiences**		508,658 (17.27)
	Vaccination disclosure	201,102 (6.83)
	Postvaccination symptoms and effects	307,556 (10.44)
**Pharma industry: vaccine development, production, and distribution**		462,529 (15.71)
	Vaccine efficacy	139,280 (4.73)
	Clinical trials, approvals, and suspensions	182,673 (6.20)
	Vaccine distribution and shortage	140,576 (4.77)
**Vaccination policies**		630,606 (21.42)
	Vaccine affordability	116,205 (3.95)
	Regulation: mandatory versus optional	410,466 (13.94)
	Travel	103,935 (3.53)
**Vaccination rollout**		176,329 (5.99)
	Vaccination appointment and scheduling	105,586 (3.59)
	Vaccination sites	70,743 (2.40)
**Attitudes toward vaccination**		1,093,050 (37.12)
	Vaccination eligibility and policies	76,605 (2.60)
	Vaccination promotion and advocacy	264,368 (8.98)
	Vaccination hesitancy	371,843 (12.63)
	Opinion leaders and endorsement	172,002 (5.84)
	Hoax/conspiracy	208,232 (7.07)
Gratitude toward health care workers		73,358 (2.49)

### Temporal Trends in Sentiments

**Figure 2:**
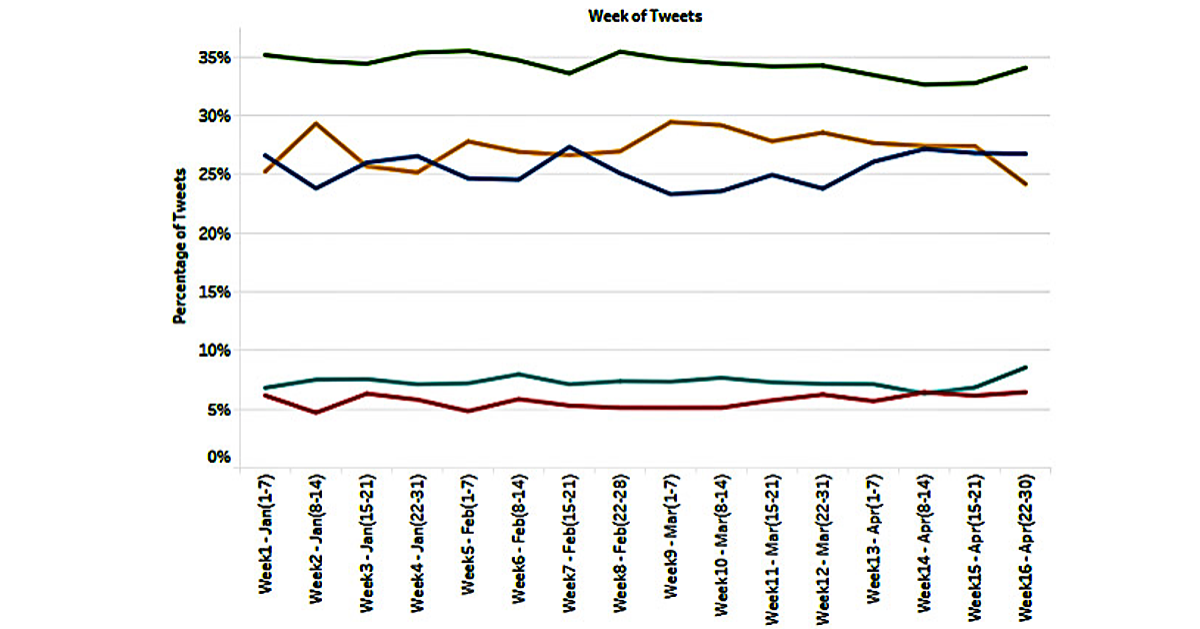
Proportions of positive, negative, and neutral tweets about COVID-19 vaccination.

We computed the sentiment scores of COVID-19 vaccination tweets and tracked their changes over the time period of our study. The results are presented in Figure 2. The proportion of positive or overly positive tweets was always greater than that of negative or overly negative tweets in all of the weeks examined. Overall, 41.62% of the tweets had a positive sentiment, 31.16% had a negative sentiment, and 27.22% had neutral sentiment scores.

We further examined the trends in sentiments of the 16 topics over time. These results are presented in Multimedia Appendix 2. A large proportion of tweets about *postvaccination symptoms and side effects* (40%-45%) and those about *conspiracy/hoax* (35%-45%) had negative or overly negative sentiments in all weeks of our examination. In contrast, greater proportions of tweets about *vaccination disclosure* (35%-40%), *vaccine efficacy* (45%-55%), *clinical trials and approvals* (30%-40%), *vaccine affordability* (35%-35%), *vaccine regulation* (30%-35%), *travel* (35-45%), *opinion leaders and endorsement* (30%-50%), and *gratitude to health care workers* (30%-45%) carried positive or overly positive sentiments throughout the time period of our research.

We also examined the trends in the average sentiment score for each of the 16 topics over the time period of examination and plotted the average compound scores by topic and week. The results are presented in Multimedia Appendix 3. Average compound sentiment scores were found to be negative throughout the time period of our examination for the following themes: *postvaccination symptoms and side effects*, *hoax/conspiracy*, and *vaccine hesitancy*. We found reversal of average sentiment scores from positive to negative during a few weeks for the topic of *vaccination policies*. For the rest of the topics, the average compound sentiment scores were consistently positive for all weeks.

### Qualitative Content Assessment

#### Overview

To further examine the public sentiments and attitudes toward COVID-19 vaccines and vaccination rollouts, we qualitatively examined the tweets for the top three themes that emerged from our topic modeling assessment.

#### Public Attitudes Toward COVID-19 Vaccine Regulation

Approximately 14% of the tweets about COVID-19 vaccination in the study period focused on the issue of whether vaccines need to be made mandatory. Many tweeters argued for mandatory vaccination, especially in places of work, schools, education institutions, and for travel:

Just like having a vaccination card to go to school, I feel businesses and all schools should make it mandatory to have Covid vaccine

Would you refuse to take the Covid vaccine; if it became compulsory to work?

If, eventually, we need to show proof of vaccination to go to theatres, restaurants, sporting events etc then no, it’s not truly optional - by any reasonable measure that’s coerced vaccination.

Tweeters also argued for making COVID-19 vaccines mandatory to health care workers. Several countries such as France have introduced mandatory vaccination requirements for health care workers. Saudi Arabia announced that all of the employees in the public, private, and nonprofit sectors must be vaccinated before they can return to work. Italy introduced a vaccination requirement for all of their health care workers and pharmacists [42]. There were many tweets that supported this type of mandatory vaccination

I support #MandatoryVaccination for nurses

Let’s keep pushing for #MandatoryVaccination of those who care for our most vulnerable Ridiculous that we're making vaccination optional for healthcare workers...vaccinate or GTFO.

Tweeters opposed to mandatory vaccination opined about how such mandates can be extended to other areas and expressed displeasure:

Its all part of the #mandatoryvaccination by coercion agenda. They are going to achieve it by: Divide and Rule -> getting the #vaccinated to blame the #unvaccinated. Threatening people with no sport events pubs etc. These narratives will grow and grow over the coming months. What happens to #MyBodyMyChoice if we’re forced into #mandatoryvaccination ? Next it will be #forced #abortion and #sterilization?

#### Vaccine Hesitancy

Approximately 12.63% of the tweets in our data set were about vaccine hesitancy that highlighted the reluctance of a set of Twitter users to receive COVID-19 vaccines. When we qualitatively examined these tweets, we found tweeters simply spelling out their stance to reject the vaccines, with many users highlighting reasons for not accepting vaccines. Promoting COVID-19 vaccines will need a clear understanding (particularly for those against COVID-19 vaccines) of whether people are willing to be vaccinated and the reasons why they are willing or unwilling to do so. We observed some common reasons cited by Twitter users for their vaccine hesitancy. Some users expressed concerns on how quickly the vaccines were developed and wondered about safety. For instance, one user tweeted “I don't trust a vaccine that was developed in such a short period of time, when we can’t even find one for so many other illnesses,” and another user tweeted “I don’t trust that jab...it’s usually years before a vaccine is ready....too rushed.. I don’t trust it.” There were others who expressed concerns about effectiveness of vaccines and if the vaccines can protect against newer strains of the virus. As one tweeter stated, “I’m not getting the vaccine. No one knows what’s in it or the long term effects of it, or if it can stop new variants.” From some other tweets, we observed public mistrust of the pharmaceutical industry, medical community, and governments:

I don't trust pharma and I won't be having any covid vaccine till it's been around for a while longer and the guinea-pigs have put it to good testing

I don’t trust this vaccine, I don’t trust the CDC, I don’t trust free donuts from Krispy Kreme (LMFAO), i don’t trust our government

Nope! Not getting the “vaccine”. I don’t trust the government nor companies who work with the government

#### Postvaccination Symptoms and Effects

Over 10% of tweets in our data set were about users sharing their experiences on symptoms and side effects of COVID-19 vaccines. Moreover, the average compound sentiment for this topic remained negative throughout the 4-month period. Twitter users shared information about the dose and their experiences subsequent to vaccination. While some users reported little or no side effects (“24 hours after my first jab of the Covid-19 vaccine, I have not observed any untoward effect from the vaccine”), others provided more detailed information on side effects and how they progressed over a period of time following the vaccination:

Had the jab at 11am yesterday and the chills & aches started at about 7pm last evening. Lots of Tylenol & fluids.

I received my 2nd covid shot yesterday morning. The biggest side effects were weakness and terrible dizziness.

Day 2 post-vaccine was no cake walk. Fever, major aches, brain fog, sore everywhere. But man am I glad I got it

Mentions of side effects were often accompanied by messages expressing elevated feelings about protection against the virus:

I had side effects from the vaccine, but that 24 hours of chills and fever was worth it to keep myself, friends, family, and my community safe.

I would much rather take 48 hours of aches and chills from the second dose of the vaccine than risk gasping for my last breath in an ICU away from family.

## Discussion

### Principal Findings

A growing number of studies have used data from social media to explore and understand public concerns and attitudes about the COVID-19 pandemic. As governments around the world are trying to tackle the pandemic through mass vaccination, it is important to uncover public opinions and attitudes toward COVID-19 vaccines. We used a repository of approximately 3 million tweets from January 2021 until the last week of April 2021 to uncover the trends in sentiments of various themes and topics pertaining to COVID-19 vaccines. We focused on tweets made by individual users and excluded those made by news outlets and other organizations. Through topic modeling, we found 16 topics pertaining to COVID-19 vaccines that were grouped into six broad themes. Further, we examined sentiments associated with these topics and the changes in sentiments over the 4-month period.

A key finding from our study is that the regulation pertaining to COVID-19 vaccines was the most discussed issue by Twitter users. The number and proportion of tweets on this theme were greater than those for all the other topics. The proportion of tweets with positive sentiments about regulation of the vaccination outweighed the proportion of negative and neutral tweets pertaining to this topic. We found vaccine hesitancy to be the second most discussed topic. We also observed negative sentiment scores for many weeks for this topic. Our qualitative analysis provided some preliminary insights into reasons behind vaccine hesitancy: shorter duration of the vaccine development cycle, concerns about effectiveness of the vaccine in controlling the virus and its variants, and general mistrust about the pharmaceutical and medical industries and governments. Another topic that was widely discussed was postvaccination side effects and symptoms. The average sentiment scores for this topic were negative throughout the time period examined.

To control the COVID-19 pandemic, it is important that a substantial portion of the worldwide population acquire immunity through vaccination. Policymakers and public health officials are increasingly focusing on ways to boost and accelerate vaccine uptake. Vaccination campaigns are being designed to address misinformation and public concerns regarding the vaccines. In addition, several efforts are being made to increase vaccine supply, introduce incentive mechanisms for encouraging vaccine uptake, and enhance public education and outreach programs. However, our findings indicate that vaccine mandates and vaccine hesitancy continue to dominate the minds of the general public, as can be seen from their posts on social media. It is important to take their attitudes into account while framing and designing vaccination campaigns and programs.

It should also be noted that most COVID-19 vaccines have been approved for emergency use and authorization, rather than through a regular licensing route. As more vaccines that are currently authorized for emergency use obtain regular approval and licenses by authorities such as the FDA, the issue of vaccine mandates is likely to gain more prominence. More employers and authorities could enforce vaccine mandates. Schools and educational institutions in many parts of the world have started mandating COVID-19 vaccines. Further, vaccination is also a requirement for most international travel. It is more likely to become a requirement for even domestic travel in several countries. A complementary approach to mandating COVID-19 vaccines is creation of trust and favorable attitudes toward vaccines in the minds of the public. Mass outreach and education programs along with incentives for vaccination can go a long way in accelerating vaccination uptake. Further, endorsement by leaders and celebrities and experience-sharing by peer individuals could also help alleviate concerns regarding vaccines.

This study points to the key issues surrounding COVID-19 vaccinations in the minds of the general public, as expressed through social media. Findings from our study bear important implications for the design of vaccination campaigns and programs. Identification of reasons for vaccine hesitancy throws light on questions that need to be answered by health policymakers and health care practitioners in order to allay the apprehensions pertaining to vaccines and their side effects. Moreover, experience sharing from the public on vaccination, side effects, and their mindsets could also serve as a morale booster for others. Some social media posts also serve as testimonials for the efficacy of vaccinations and their effectiveness. Future vaccination drives and campaigns can take into account the experiences of a fairly large body of individuals to design appropriate responses to increase vaccination uptake.

### Limitations and Future Work

This study used tweets posted from January 1 to April 31, 2021. Vaccination efforts accelerated in several parts of the world shortly after (June-July of 2021), which have not been captured by our study. It should also be noted that we used a machine learning classifier to separate tweets made by individuals and exclude those made by organizations and news outlets. This helped us to remove numerous tweets made by media outlets and organizations so that we could capture the attitudes of the general public. The classifier exhibited an accuracy of 91.81%, which is comparable or better than those reported in many other studies [35,43,44]. Given the large number of tweets as well as Twitter users in our data set, we did not specifically examine if any set of users acted as influencers or “supertweeters.” Examining the tweets of celebrities or other influencers could help to uncover the impacts of these influencers on vaccination campaigns as a potentially fruitful area of future work.

Another limitation is that we covered only tweets posted in the English language. Due to the nature of the data we gathered, we did not explore any geographical disparities in the tweets, which could also be a fruitful extension to our work. Another extension of our work would be to examine emotions expressed in tweets pertaining to COVID-19 vaccinations. Another important limitation of our study is that we have captured only the attitudes and opinions of Twitter users, who have a presence in social media. Twitter users tend to be technology-savvy, adept in using social media, and own smartphones, and therefore may not represent the entire population set. A larger set of the population who do not have a presence on Twitter has not been covered by our study.

### Conclusion

With variants of the virus causing COVID-19 creating multiple waves of the pandemic in several countries, it is important to accelerate the rate of vaccinations and improve uptake. As COVD-19 vaccination efforts move forward, it will be important to continue to monitor public opinions regarding vaccine mandates, vaccine hesitancy, and vaccination uptake. Some individuals and groups are likely to continue to oppose vaccines, whereas there may be many others who could be convinced by appropriate education and outreach programs. While mandates by governments or employers could be contested on legal grounds, appropriate exemptions will need to be made for people with certain health conditions or special situations. Infoveillance based on social media data can provide rich insights for policymakers and health officials to frame appropriate policies and programs for COVID-19 vaccination.

## Appendix

Multimedia Appendix 1 Themes, topics, and keywords.

Multimedia Appendix 2 Trends in sentiments of tweets.

Multimedia Appendix 3 Average sentiment scores and trends.

## Abbreviations

CorEx: Correlation Explanation
FDA: Food and Drug Administration
LDA: latent Dirichlet allocation
VADER: valence aware dictionary and sentiment reasoner
