# Supplementary material for: Examining Public Sentiments and Attitudes Toward COVID-19 Vaccination: Infoveillance Study Using Twitter Posts
Source: JMIR Infodemiology. 2022 Apr 15;2(1):e33909. doi: 10.2196/33909 (PMC9014796; doi:10.2196/33909)
Supplement: Multimedia Appendix 1 [file infodemiology_v2i1e33909_app1.docx]

**Appendix 1: Themes, Topics and Associated Keywords**

| **Themes and Topics** | **Top Keywords** |
| --- | --- |
| 1. Vaccination Experiences |  |
| 1.1 Vaccination disclosure | shot, jabbed, got, vaccinated, fully vaccinated, first dose, second dose, today |
| 1.2 Post-vaccination symptoms  and effects | symptom, fever, chills, pain, fatigue, headache, nausea, vomiting, sore, swollen, tired, sleepy, sick, dizziness, numbness, reaction, ache, flu |
| 2. Pharma industry: Vaccine Development and Production |  |
| 2.1 Vaccine efficacy | efficacy, effective, compared, efficient, survive, accurate, protects, immune, immunity |
| 2.2 Clinical trials, approvals and  suspensions | approval, clinical, trial, rollout, release, study, experiment, emergency, authorized, lawful, regulatory, breakthrough, suspend, license, ban, temporary. |
| 2.3 Vaccine distribution and  shortage | distribution, production, logistics, manufacture, export, import, million doses, billion doses, transport, backlog, shortage, scarcity, stock, limited, insufficient, deficit |
| 3. National Policies on Vaccination |  |
| 3.1 Vaccine Affordability | free, costly, taxpayer, capped, fee, profits, paid, expensive, gouge |
| 3.2 Regulation -   Mandatory vs Optional | optional, required, necessary, protocol, enforce, regulation, certificate, compulsory, obligatory, forced, mandate |
| 3.3 Travel | passport, travel, airline, plane, airfare, visa, flight, train, airport, trip, hotel, vacation, border, sealed, fly, vaccine passport |
| 4. Vaccination Rollout |  |
| 4.1 Vaccination Appointment  and Scheduling | book, schedule, website, email, walk-in, appointment, register, hotline, reschedule, cancellation, drive-thru, sign-up |
| 4.2 Vaccination Sites | location, miles, drive, county, hospital, far, site, clinic, pharmacy, centers, community |
| 5. Attitudes towards Vaccination |  |
| 5.1 Vaccination eligibility and  policies | eligible, resident, senior, old, elderly, age, policy, under, young, eligibility |
| 5.2 Vaccination promotion and  advocacy | getvaccinated, readytovaccinate, vaccineswork, vaccinessavelives, saves, advocacy, thisisourshot, urge, vaccinate, vaccinatethem, vaccineforall, encourage |
| 5.3 Vaccination hesitancy | antivaccine, vaccinekills, novaccination, nomandatoryvaccine, justsayno, novaccineforme, boycottvaccine, saynotovaccines, boycott, danger, sayno, nogood, novaccine, refuse, hesitant, vaccinehesitancy |
| 5.4 Opinion leaders and  endorsement | Trump, biden, modi, trudeau, joe, desantis, realdonaldtrump, olaf scholz, cuomo, fauci, ministry, president, congress, republican, democrat, potus, celebrity, actor, sports, governor, government, minister, mayor, secretary, leader |
| 5.5 Hoax/conspiracy | fake, steal, hoax, conspiracy, misinformation, scam, fraudulent, forged, rumour, myth |
| 6. Gratitude to healthcare  workers | Grateful, healthcare, doctor, nurse, frontline, worker, volunteer, military, physician, staff, thank you |
