# Supplementary material for: Examining Public Sentiments and Attitudes Toward COVID-19 Vaccination: Infoveillance Study Using Twitter Posts
Source: JMIR Infodemiology. 2022 Apr 15;2(1):e33909. doi: 10.2196/33909 (PMC9014796; doi:10.2196/33909)
Supplement: Multimedia Appendix 2 [file infodemiology_v2i1e33909_app2.docx]

**Multimedia Appendix 2: Trends in Proportions of Positive, Neutral and Negative Tweets by Topics**

Color legend: green=positive, red=negative, yellow=neutral, teal = overly positive, orange = overly negative.

| 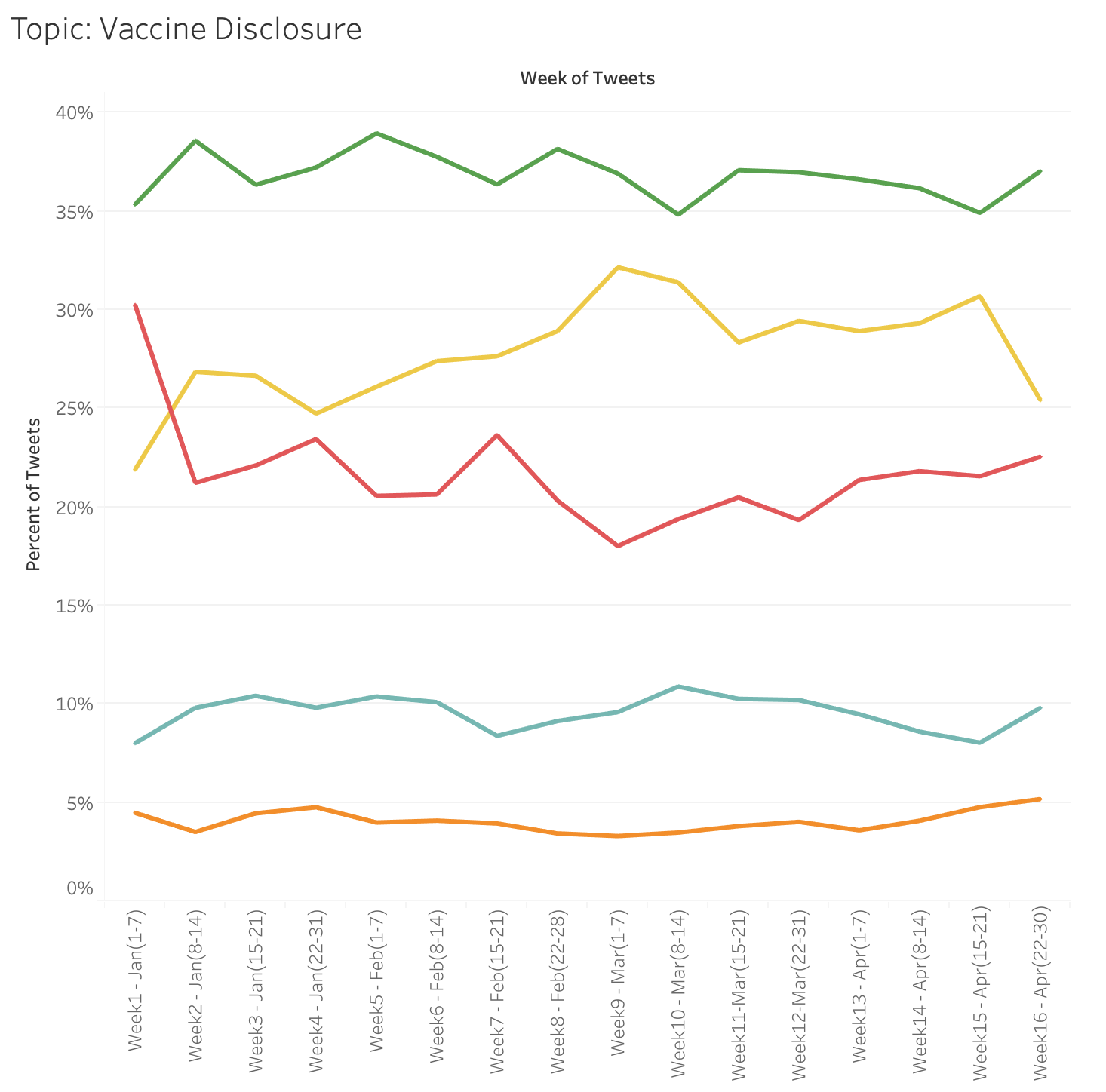 | 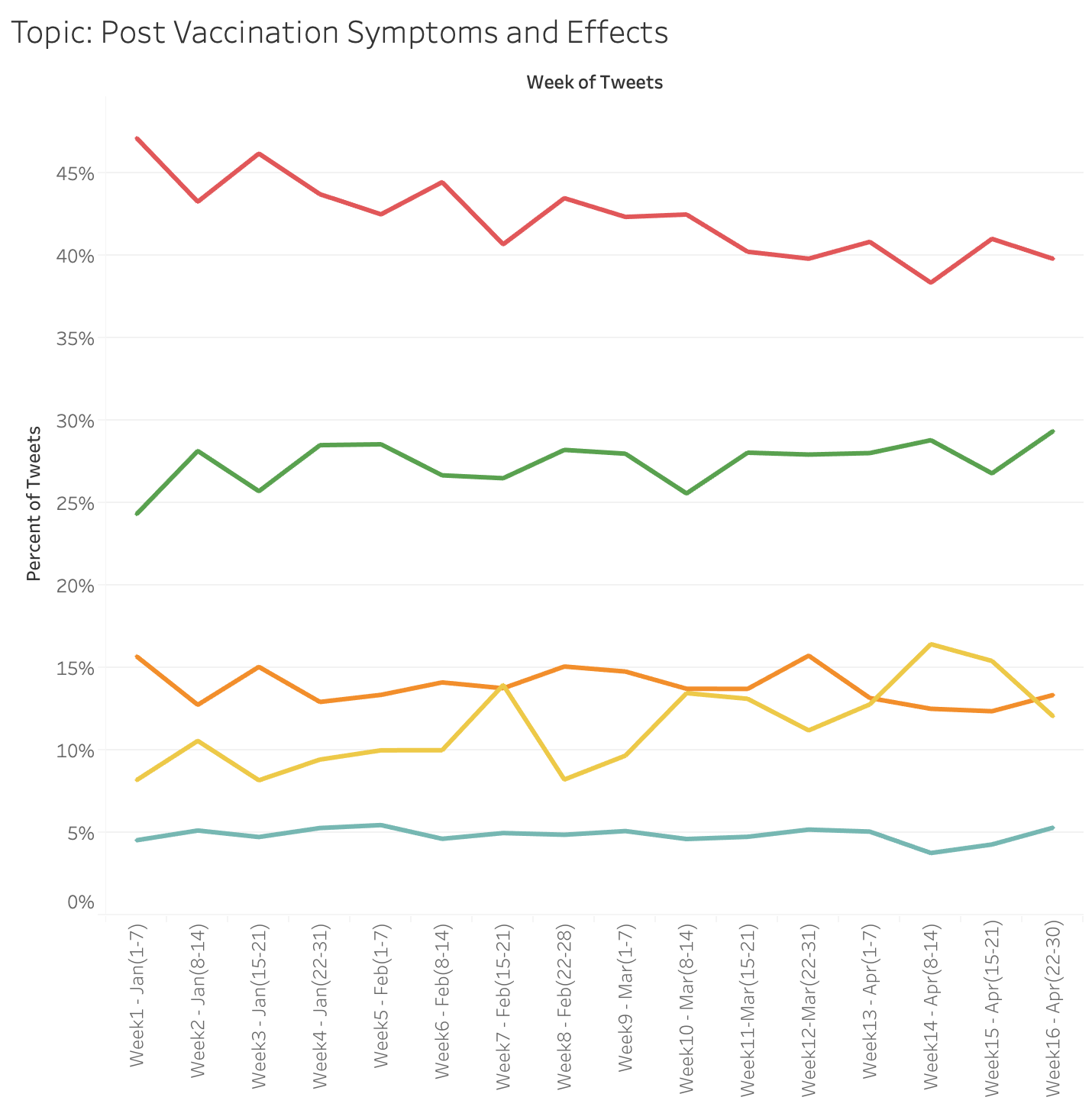 | 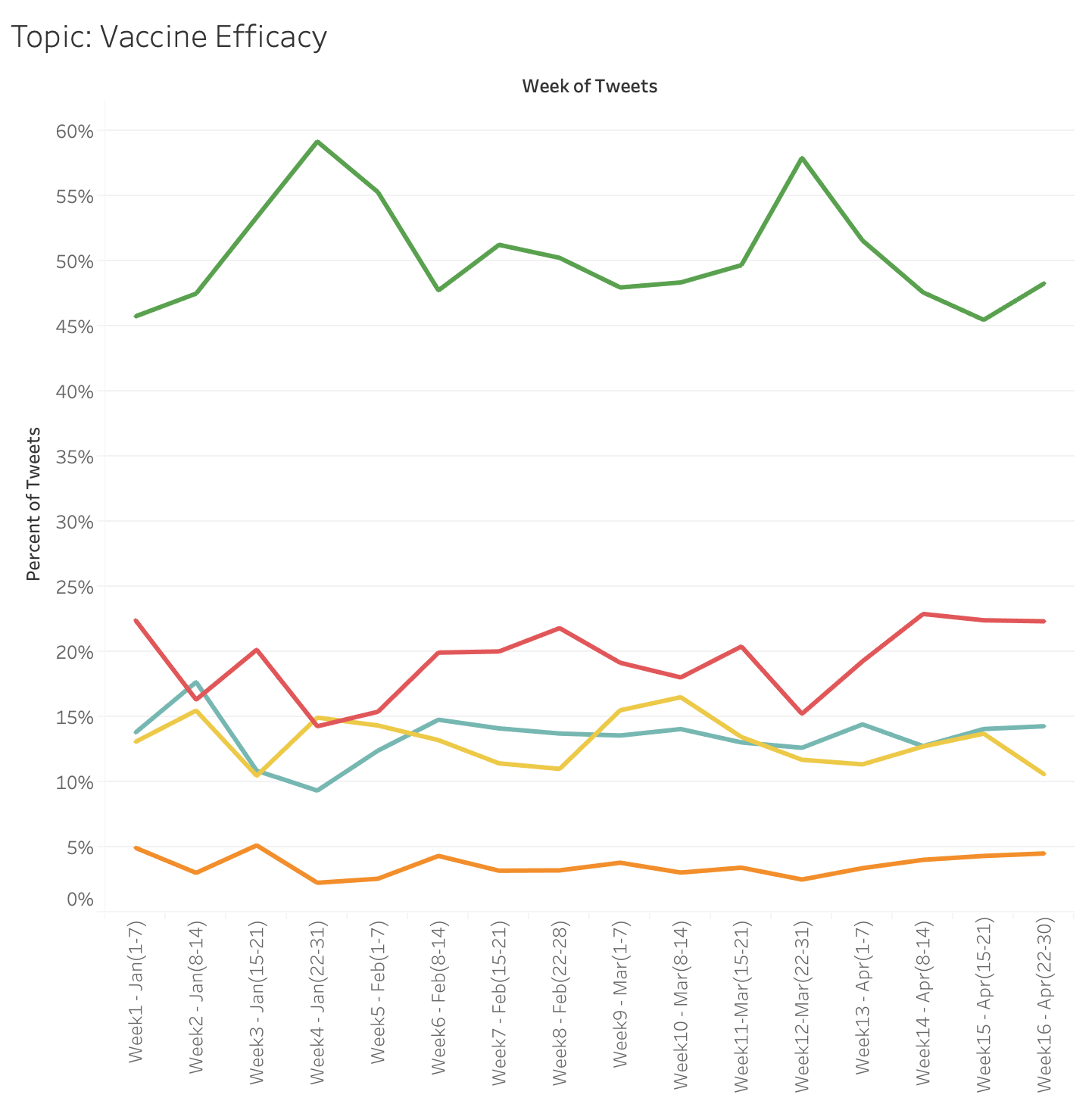 | 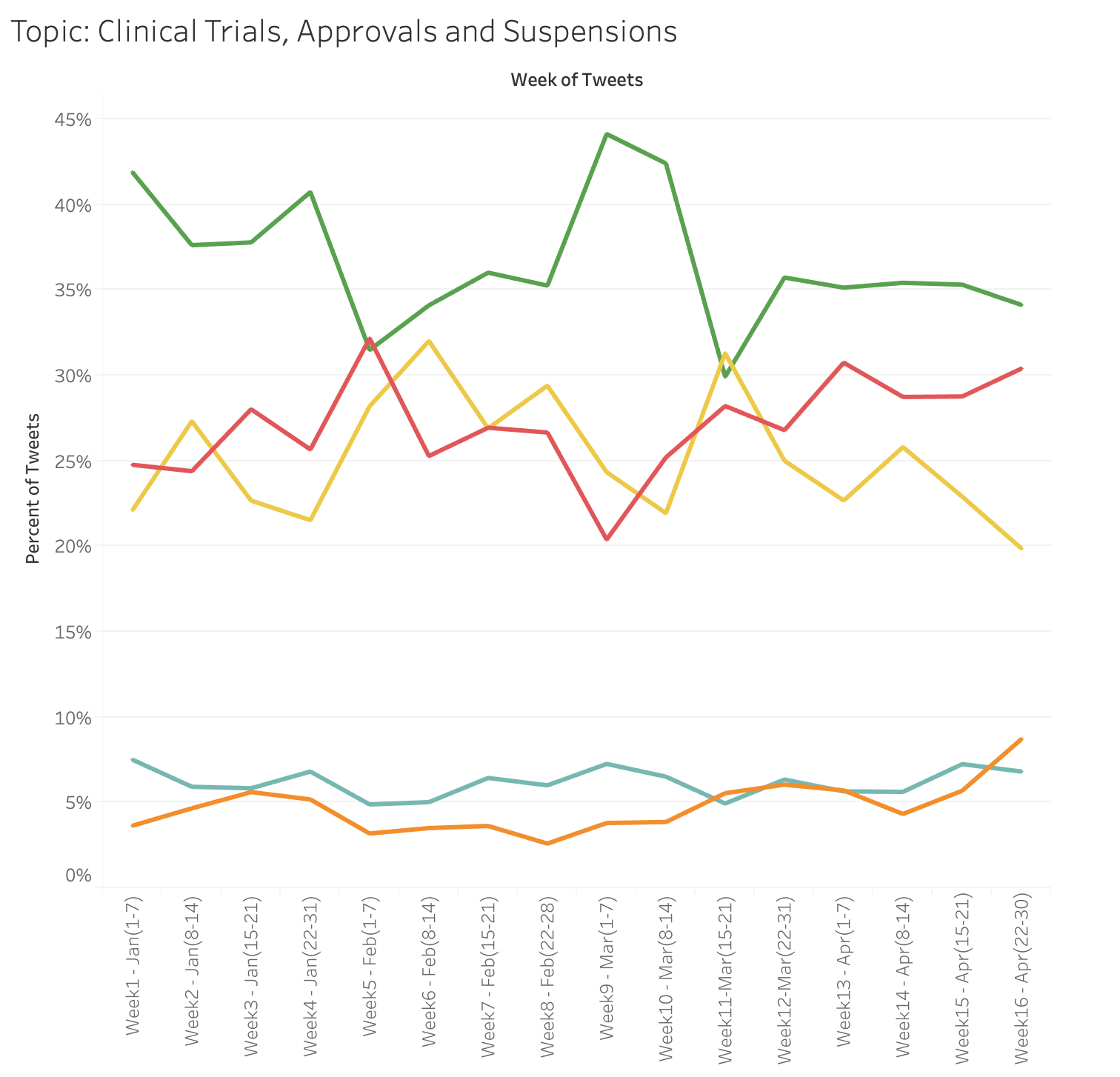 |
| --- | --- | --- | --- |
| 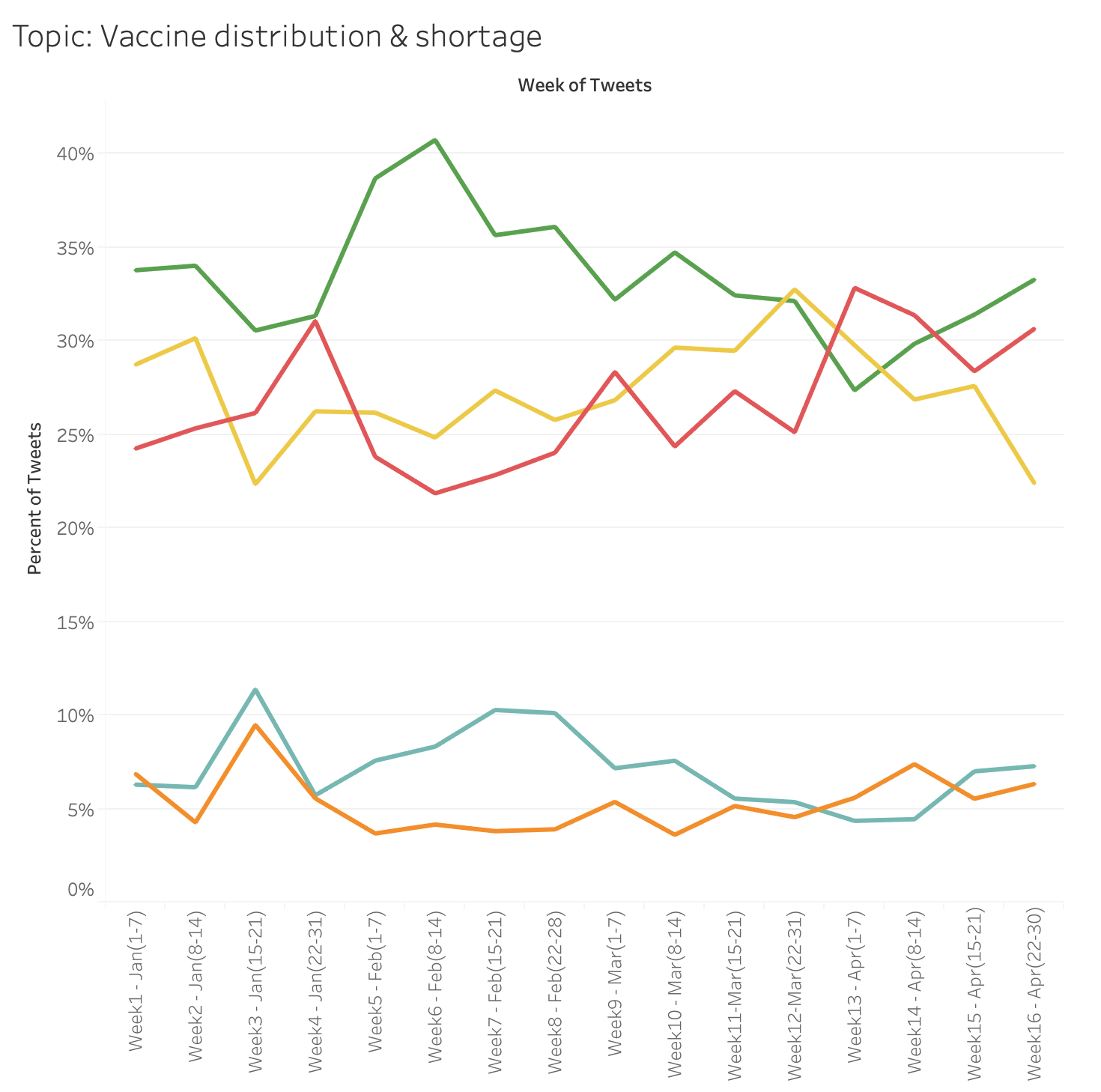 | 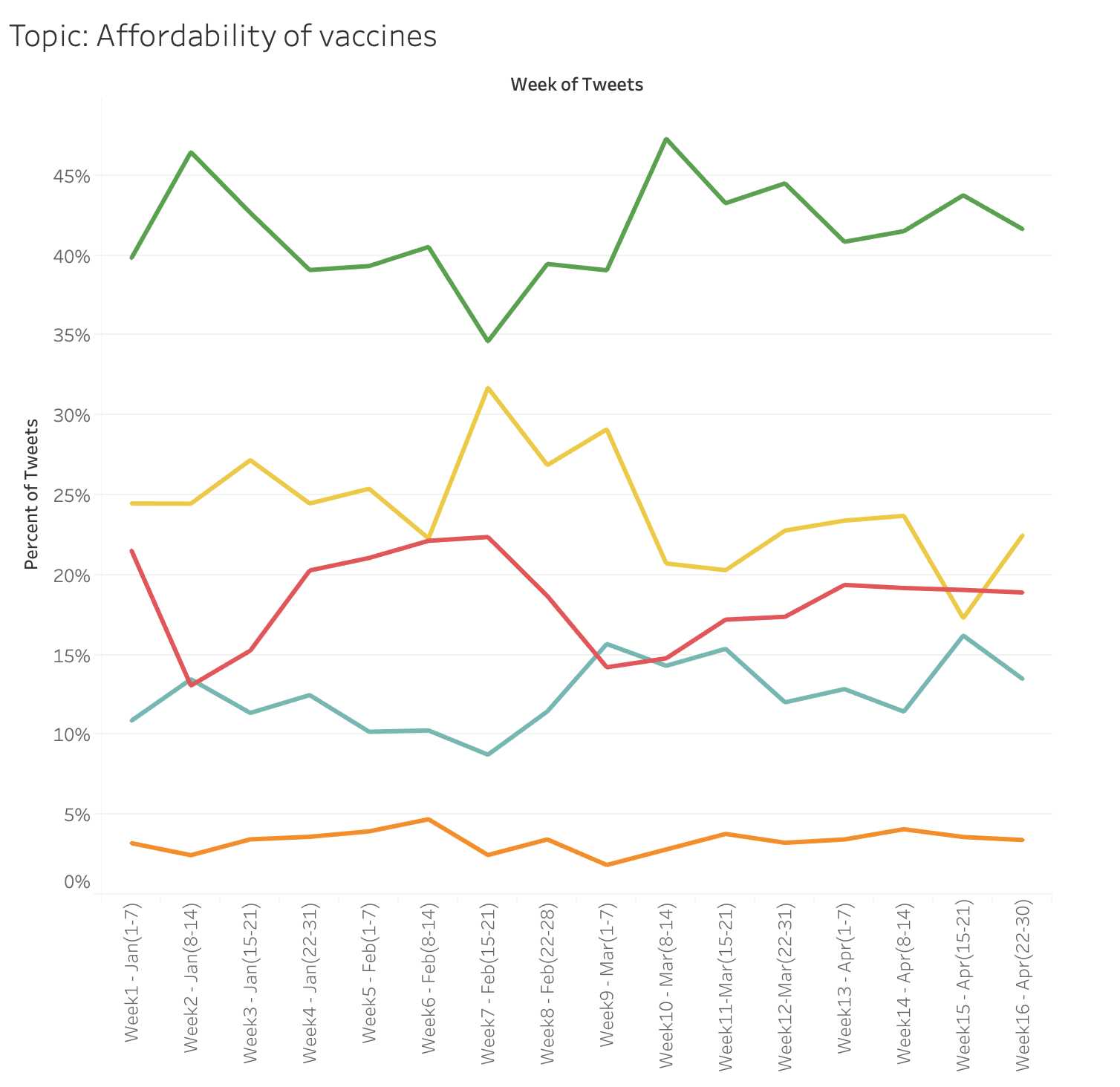 | 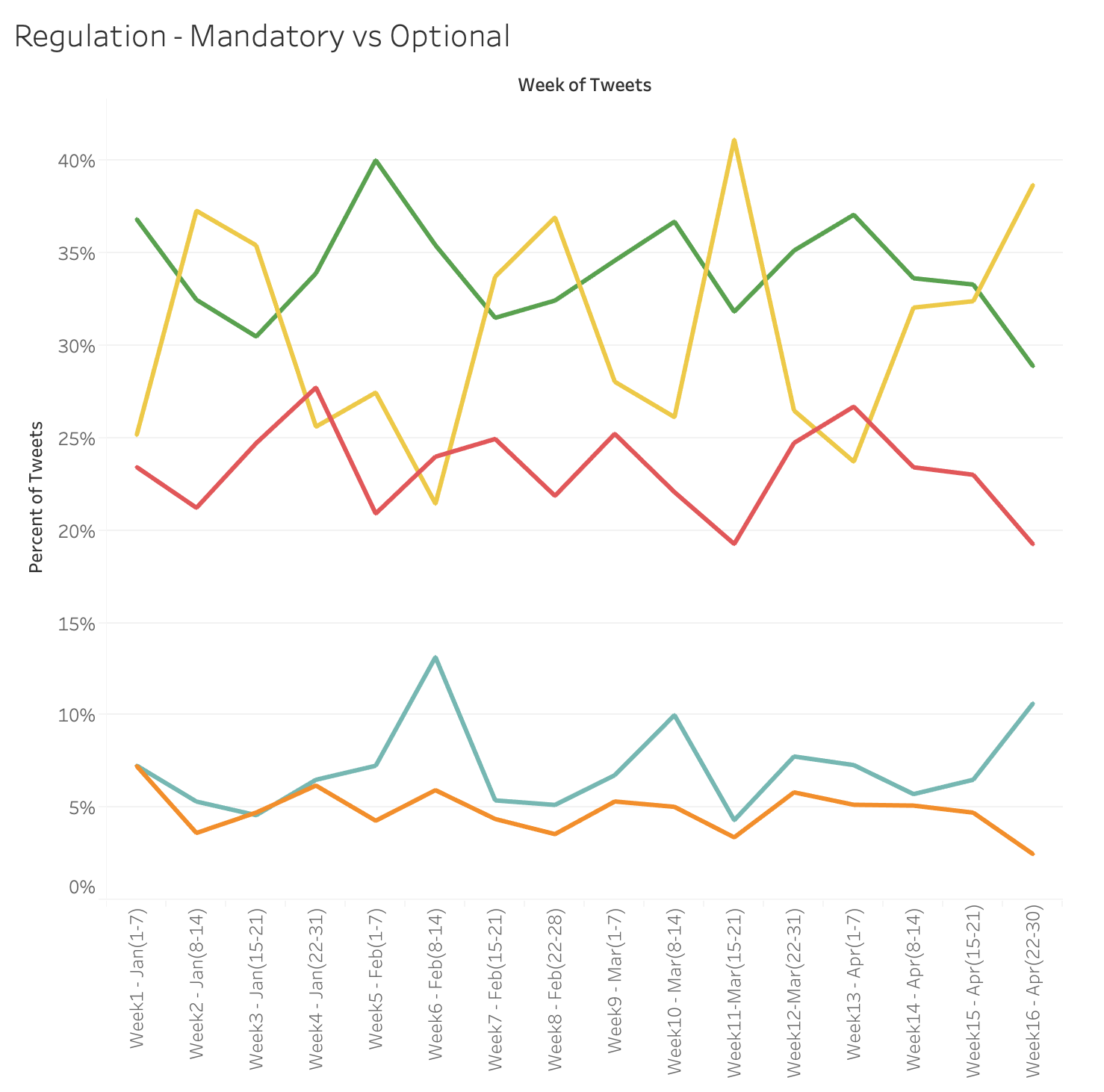 | 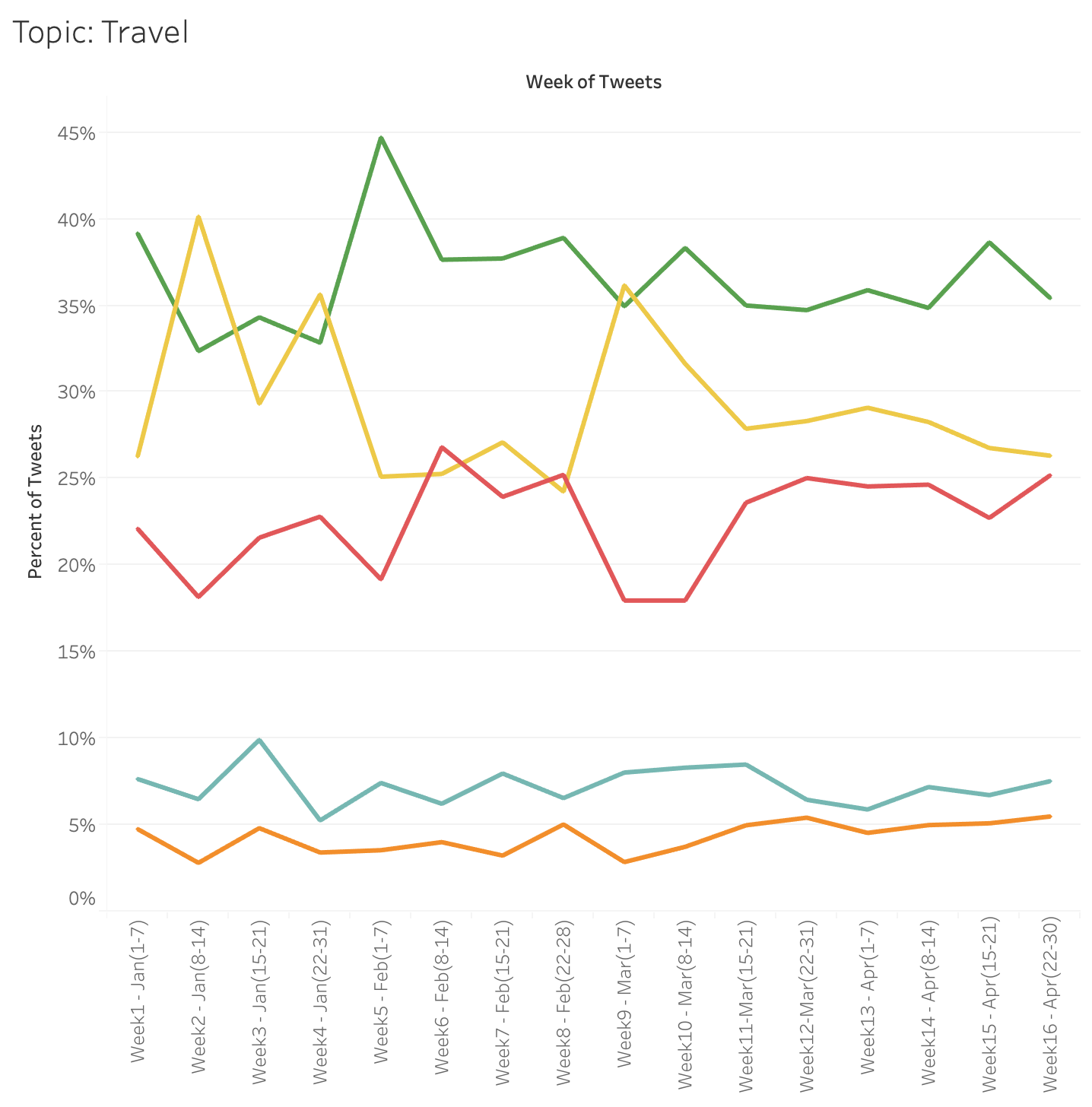 |
| 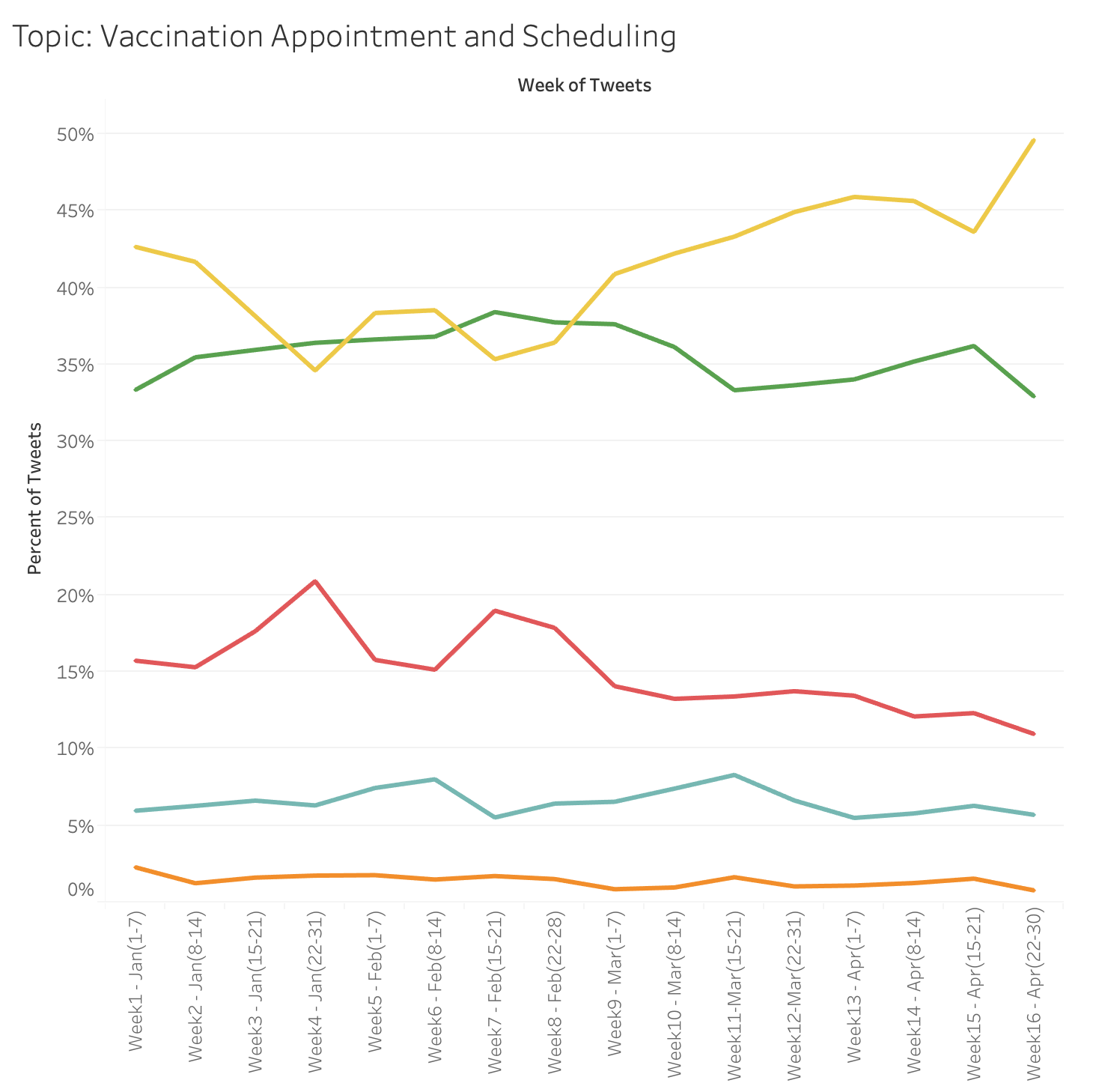 | 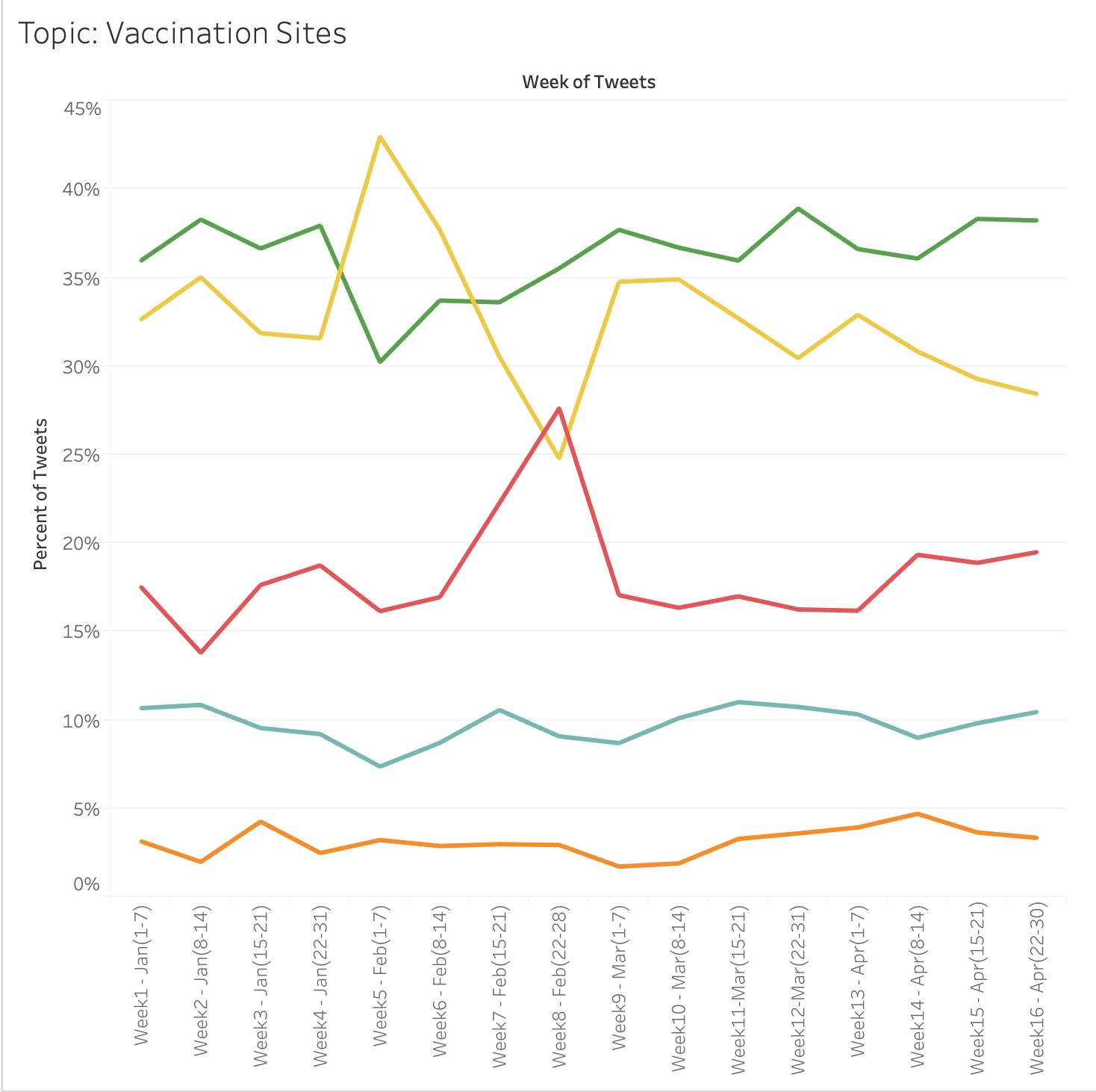 | 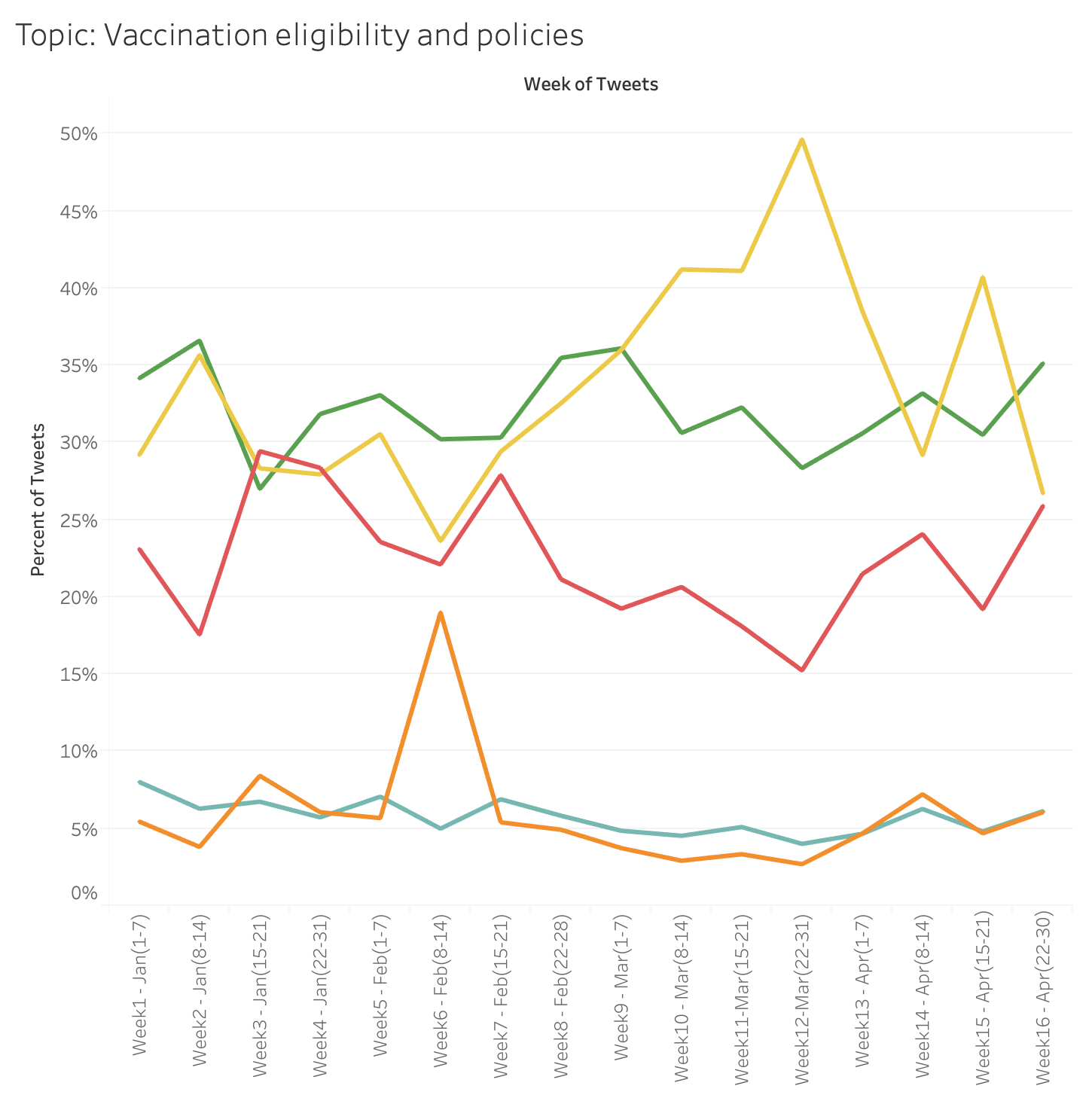 | 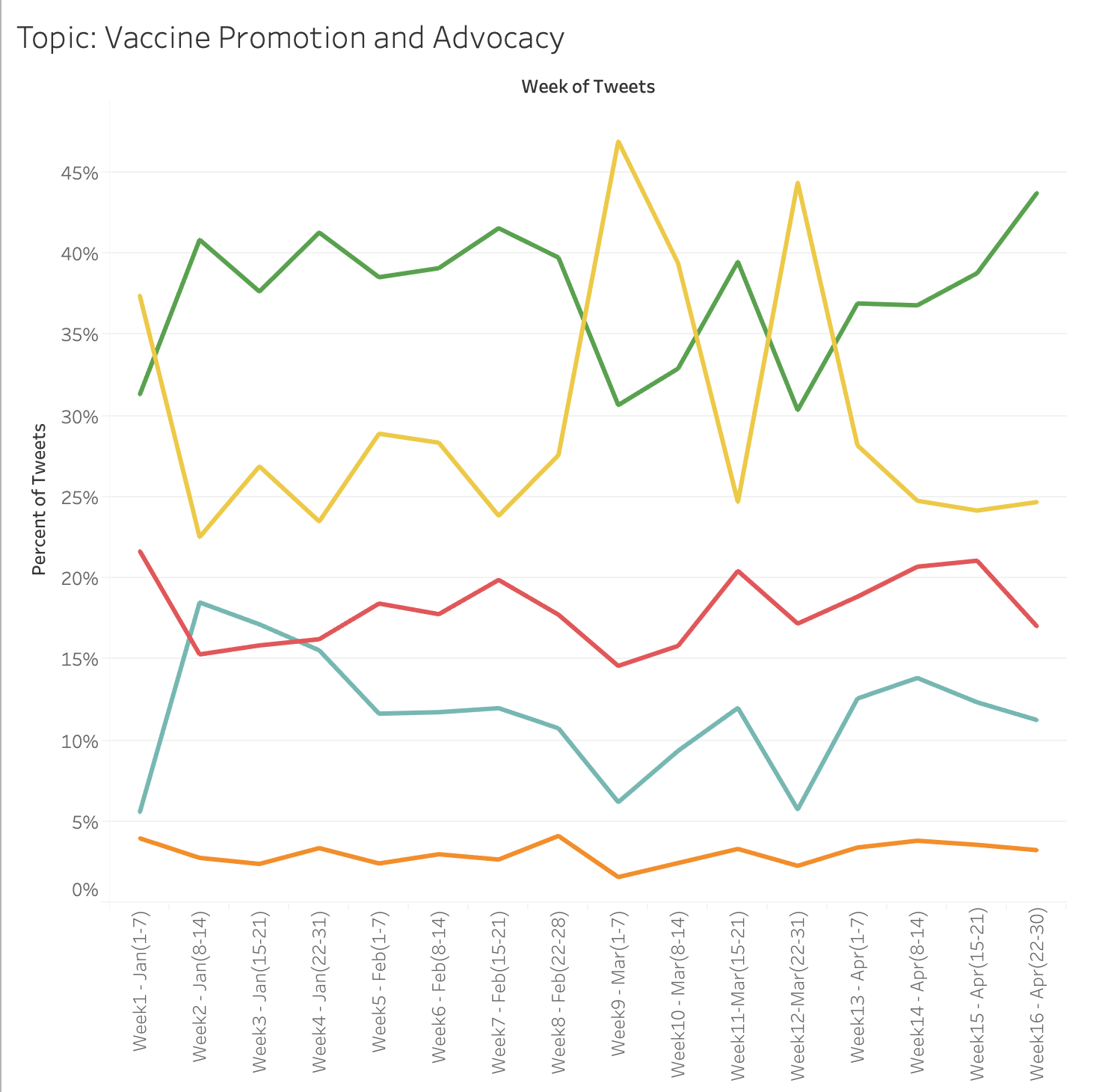 |
| 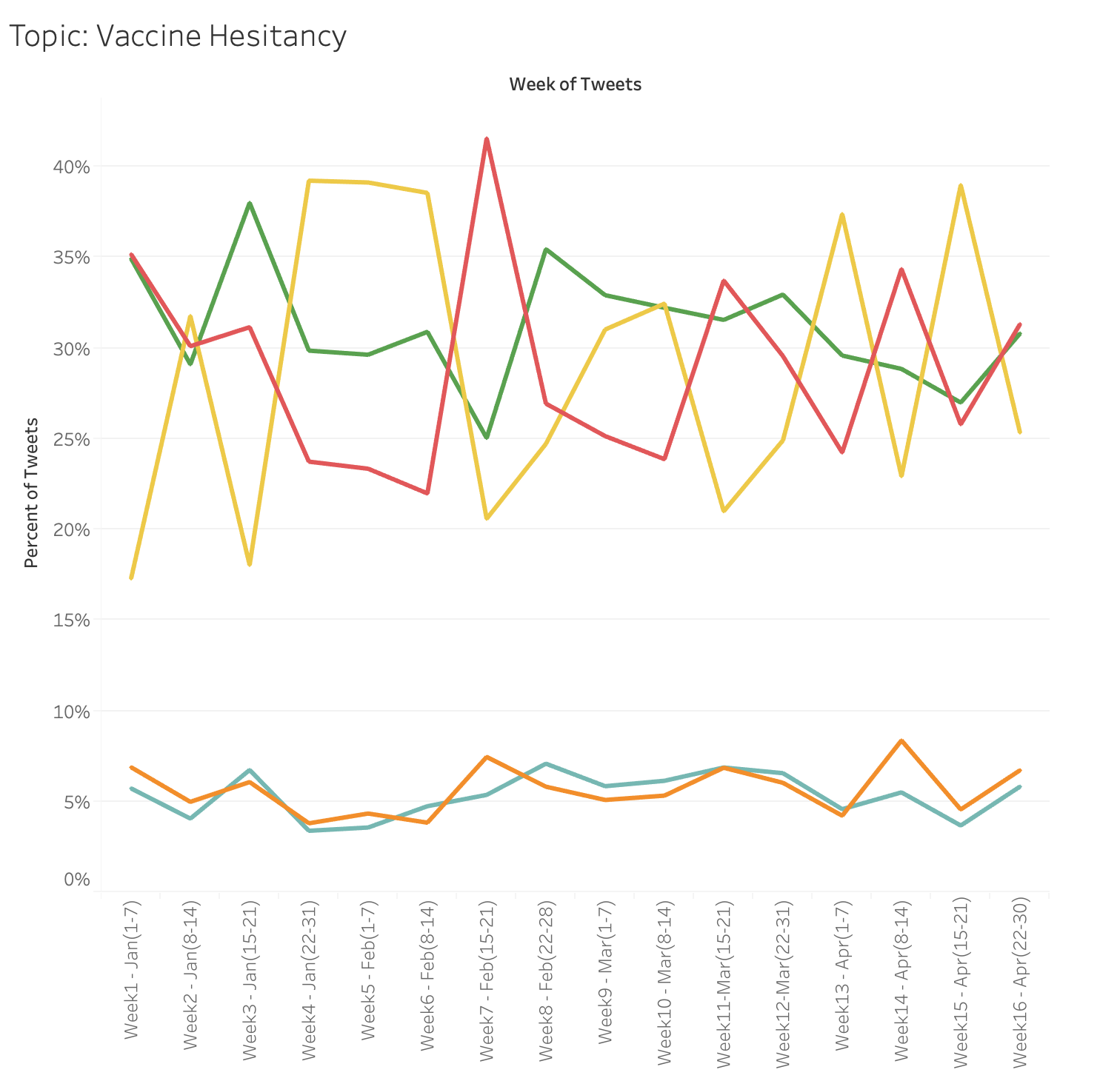 | 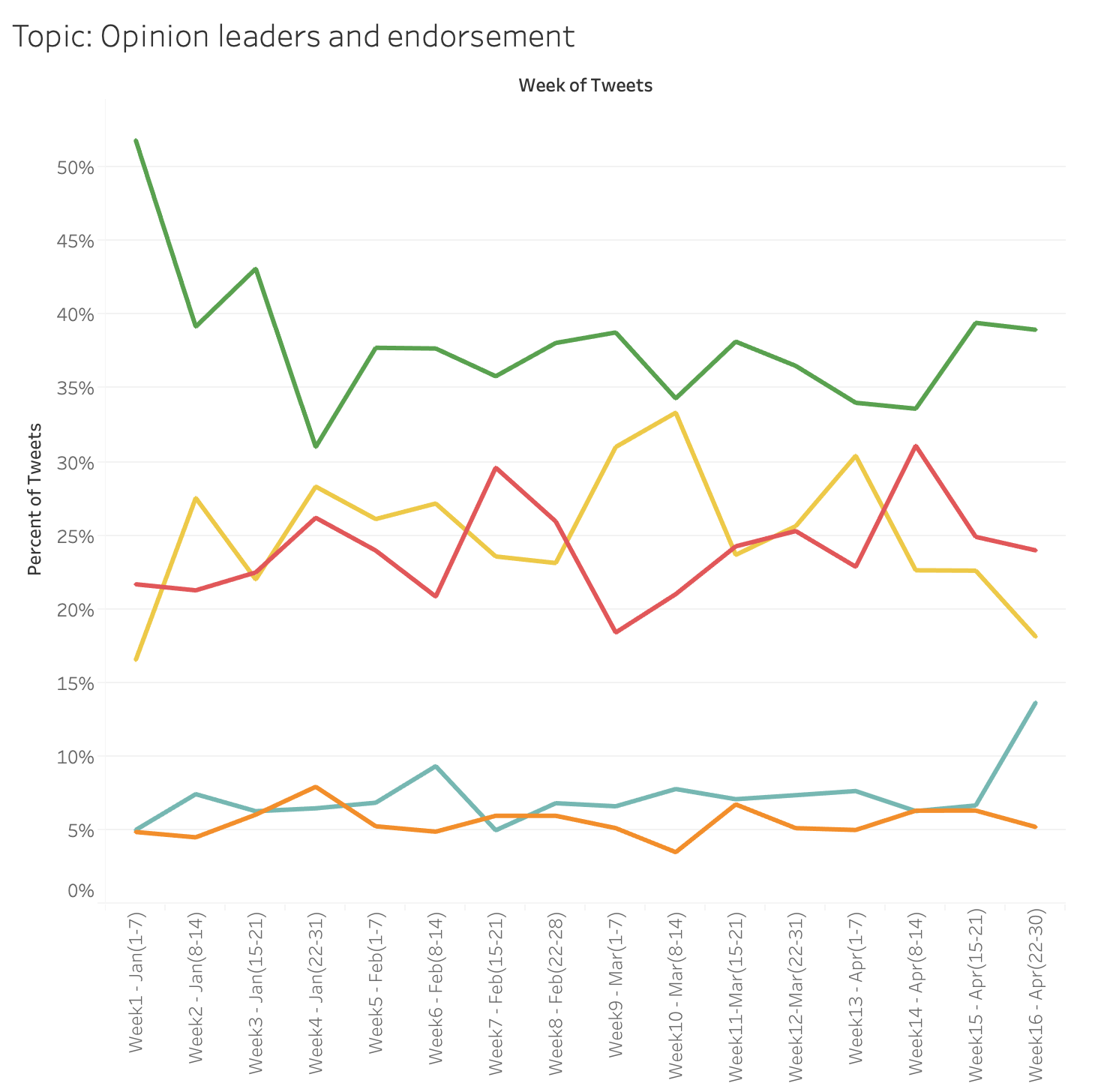 | 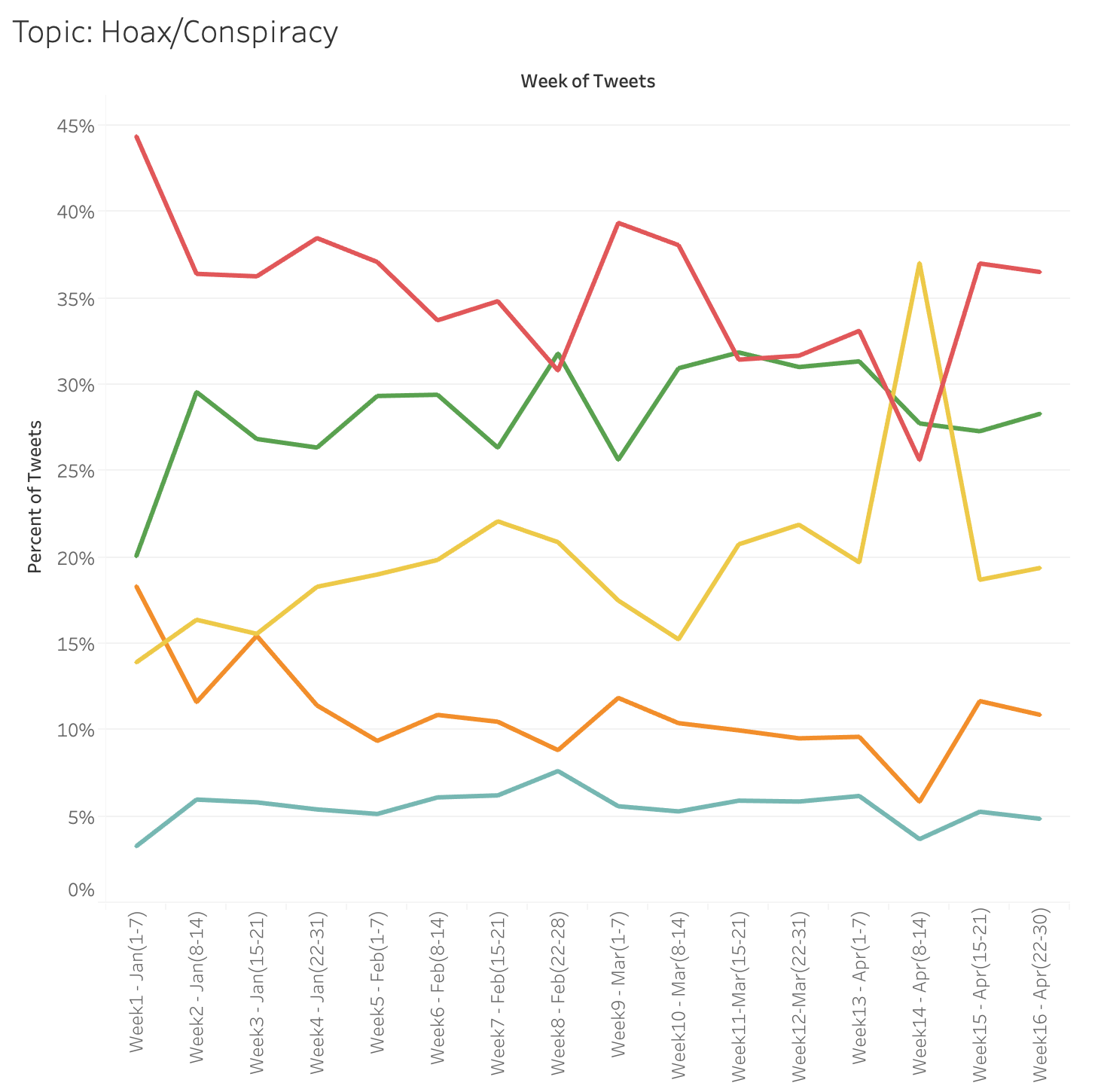 | 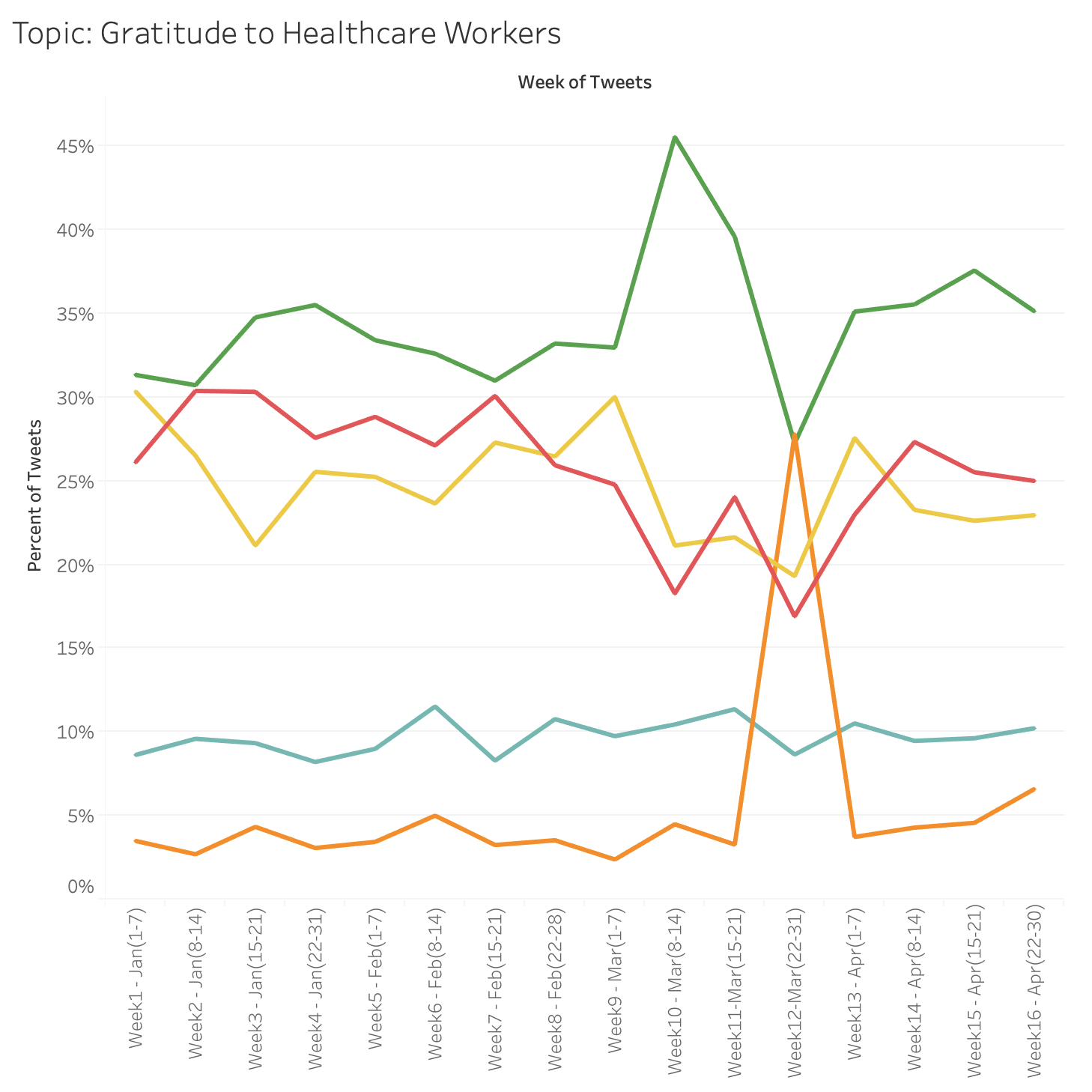 |
