# Supplementary material for: Examining Public Sentiments and Attitudes Toward COVID-19 Vaccination: Infoveillance Study Using Twitter Posts
Source: JMIR Infodemiology. 2022 Apr 15;2(1):e33909. doi: 10.2196/33909 (PMC9014796; doi:10.2196/33909)
Supplement: Multimedia Appendix 3 [file infodemiology_v2i1e33909_app3.docx]

**Multimedia Appendix 3: Average Sentiment Scores and Trends by Topics**

| 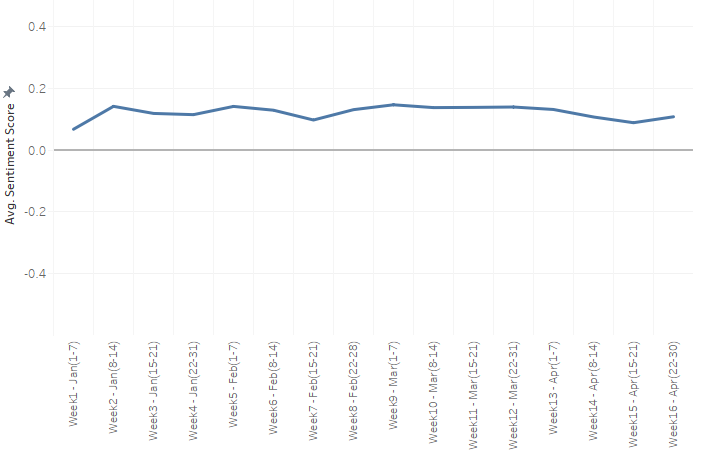  Topic: Vaccine Disclosure (n=201,102) | 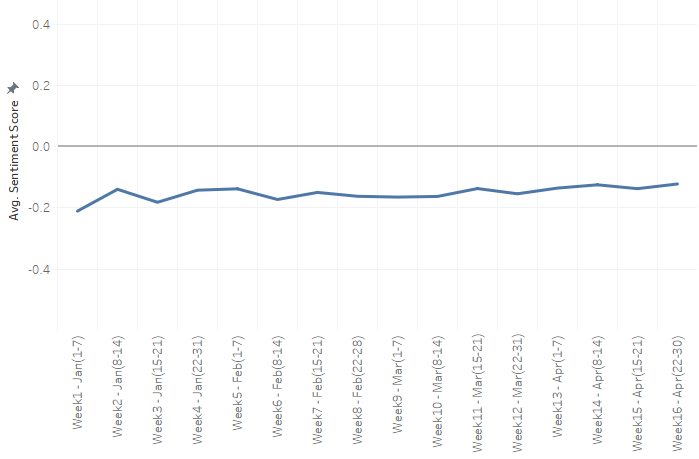  Topic: Post Vaccination Symptoms and Effects (n=307,556) | 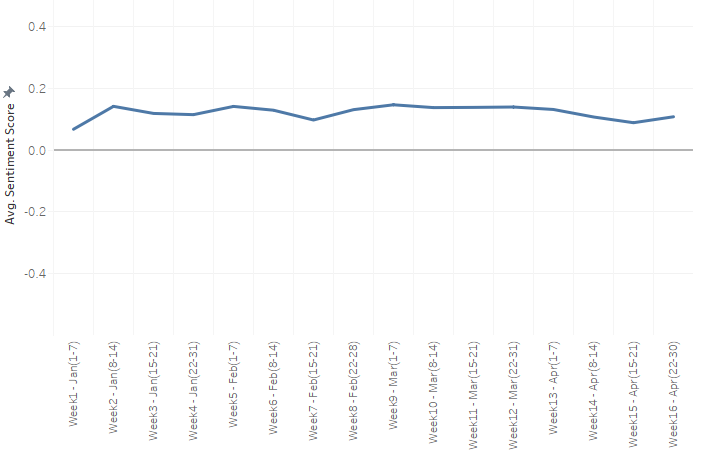  Topic: Vaccine Efficacy (n=139,280) | 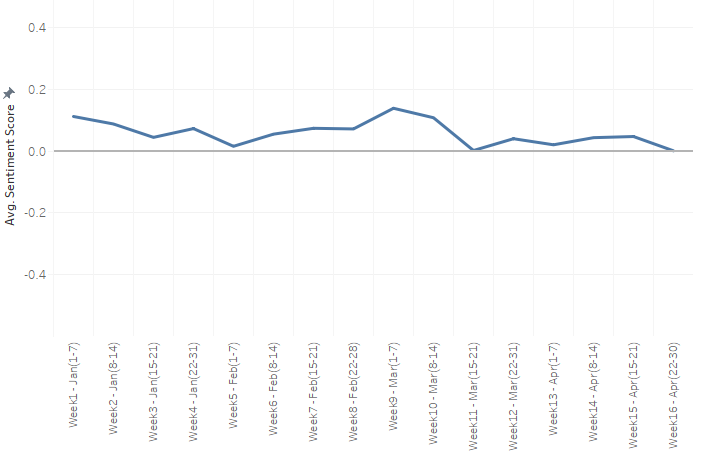  Topic: Clinical Trials, Approvals and Suspensions (n=182,673) |
| --- | --- | --- | --- |
| 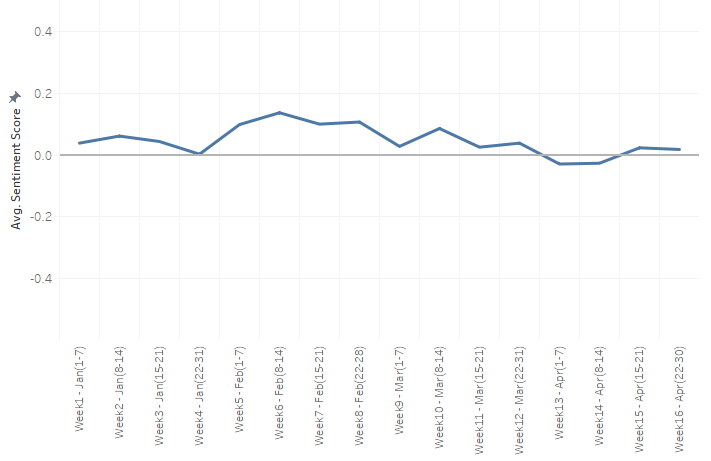  Topic: Vaccine Distribution & Shortage (n=140,576) | 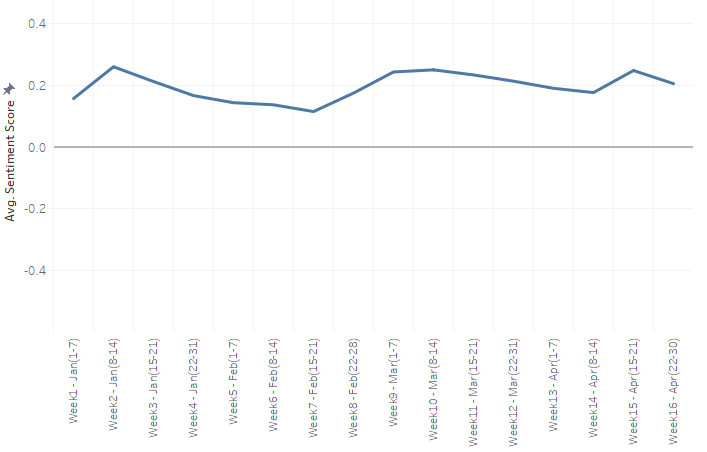  Topic: Affordability of Vaccines (n=116,205) | 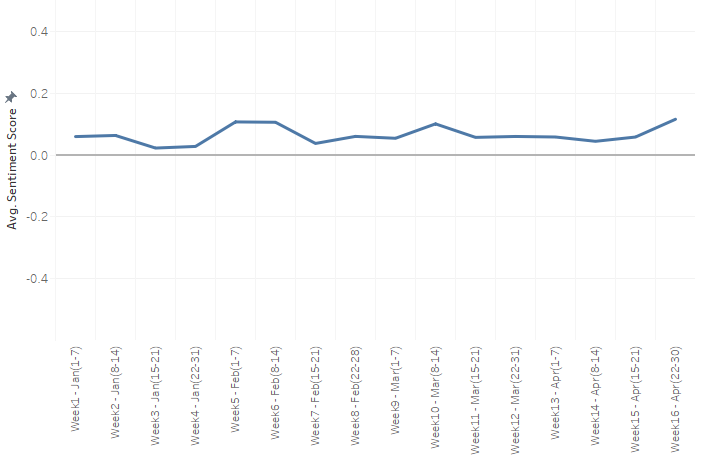  Topic: Regulation - Mandatory vs Optional (n=410,466) | 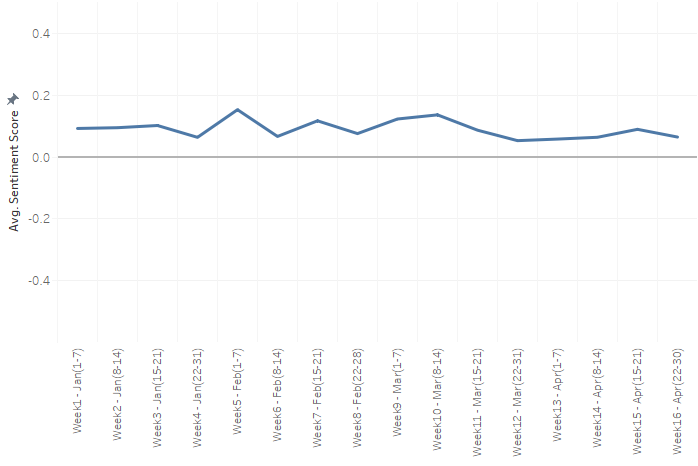  Topic: Travel (n=103,935) |
| 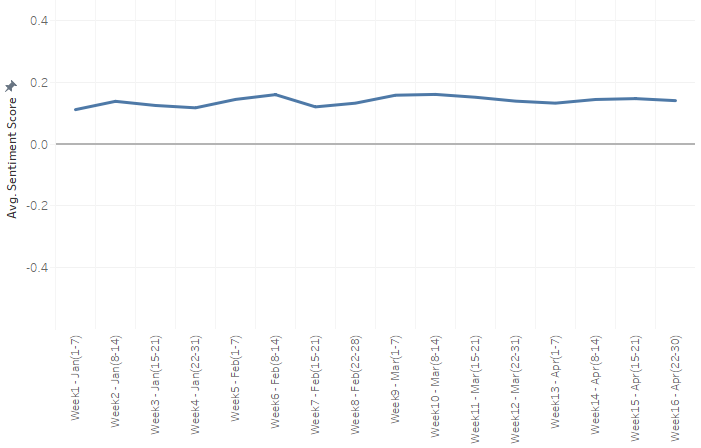  Topic: Vaccine Appointment & Scheduling (n=105,586) | Topic: Vaccination Sites (n=70,743)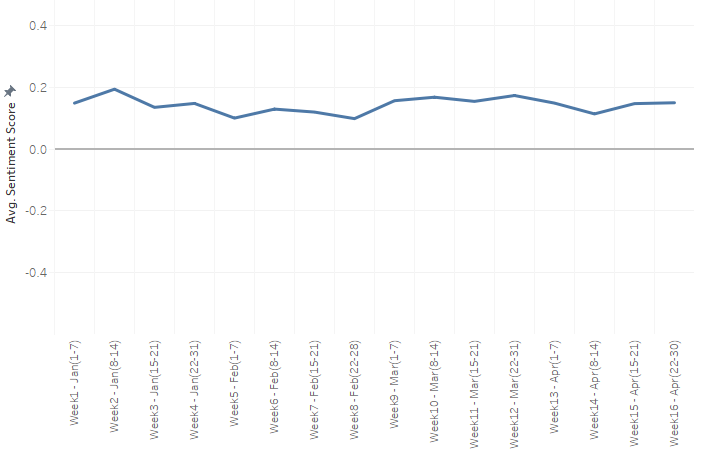 | 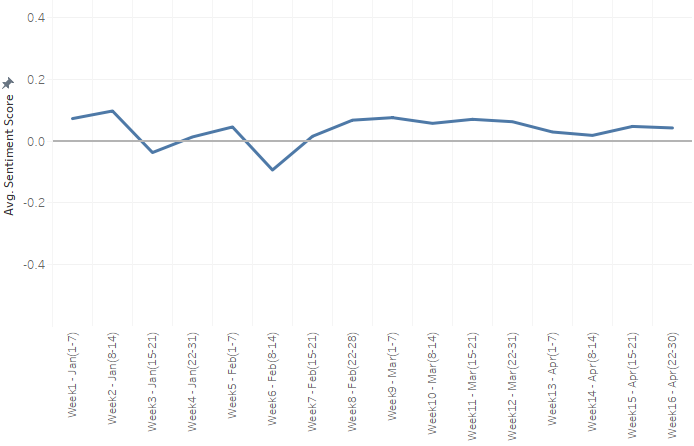  Topic: Vaccine Eligibility Policies (n=76,605) | 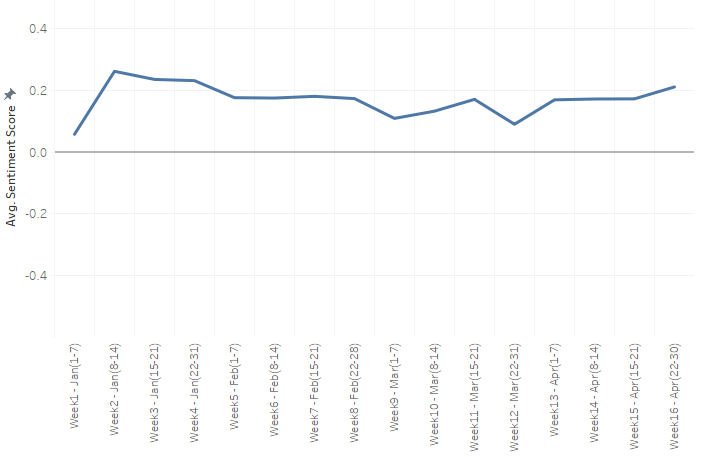  Topic: Vaccine Promotion & Advocacy (n=264,368) |
| 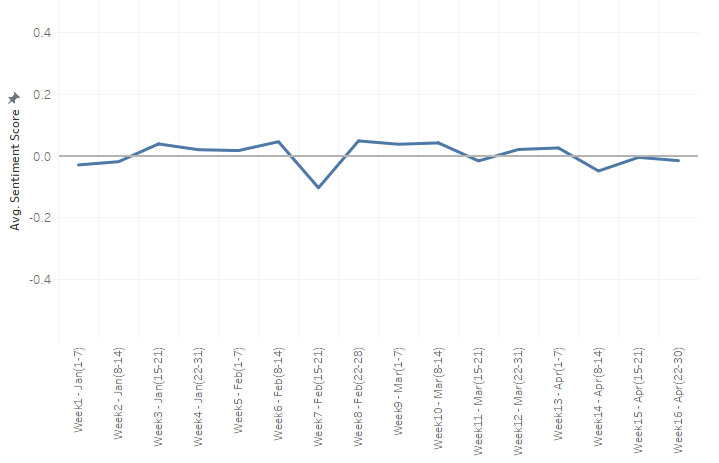  Topic: Vaccine Hesitancy (n=371,843) | 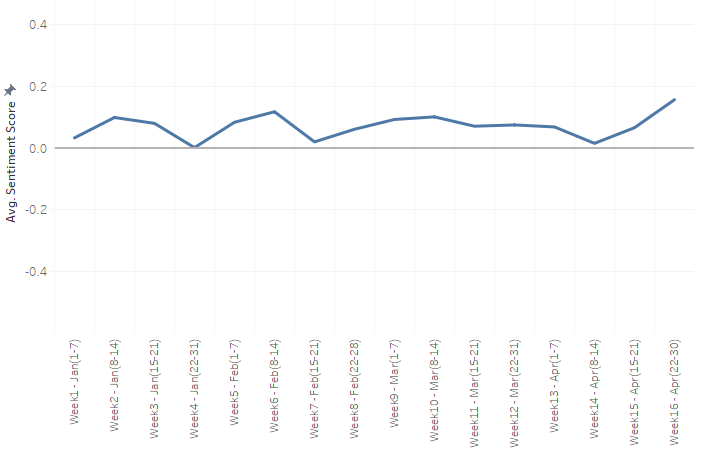  Topic: Opinion Leaders & Endorsements (n=172,002) | Topic: Hoax/Conspiracy (n=208,232)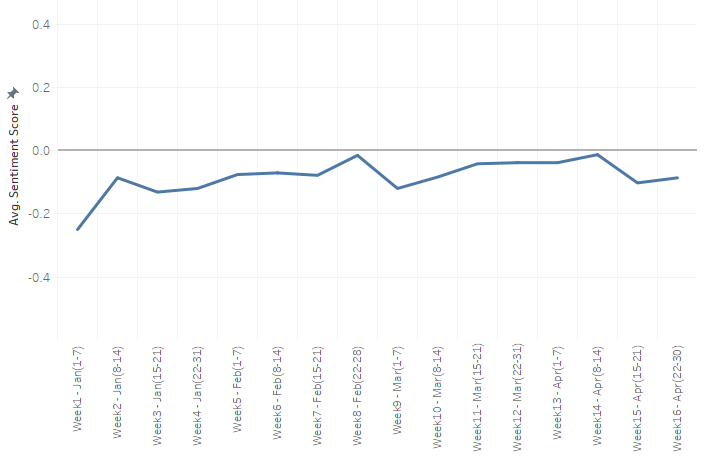 | 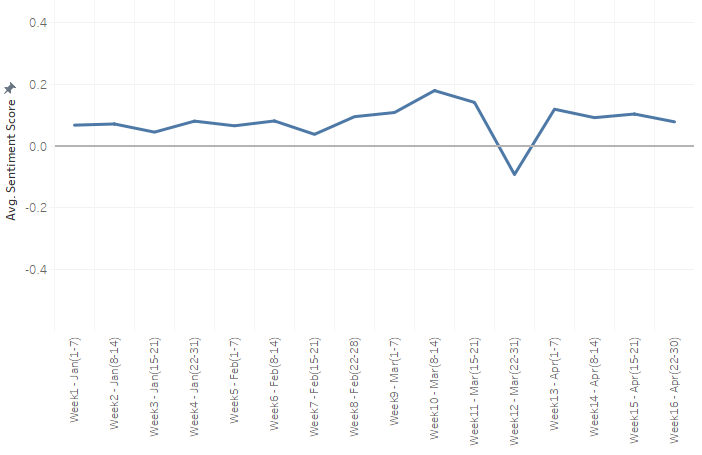Theme: Gratitude to Healthcare Workers (n=73,358) |
